# Computational Prediction and Molecular Characterization of an Oomycete Effector and the Cognate *Arabidopsis* Resistance Gene

**DOI:** 10.1371/journal.pgen.1002502

**Published:** 2012-02-16

**Authors:** Sandra Goritschnig, Ksenia V. Krasileva, Douglas Dahlbeck, Brian J. Staskawicz

**Affiliations:** Department of Plant and Microbial Biology, University of California Berkeley, Berkeley, California, United States of America; University of Toronto, Canada

## Abstract

*Hyaloperonospora arabidopsidis (Hpa)* is an obligate biotroph oomycete pathogen of the model plant *Arabidopsis thaliana* and contains a large set of effector proteins that are translocated to the host to exert virulence functions or trigger immune responses. These effectors are characterized by conserved amino-terminal translocation sequences and highly divergent carboxyl-terminal functional domains. The availability of the *Hpa* genome sequence allowed the computational prediction of effectors and the development of effector delivery systems enabled validation of the predicted effectors in *Arabidopsis*. In this study, we identified a novel effector ATR39-1 by computational methods, which was found to trigger a resistance response in the *Arabidopsis* ecotype Weiningen (Wei-0). The allelic variant of this effector, ATR39-2, is not recognized, and two amino acid residues were identified and shown to be critical for this loss of recognition. The resistance protein responsible for recognition of the ATR39-1 effector in Arabidopsis is RPP39 and was identified by map-based cloning. RPP39 is a member of the CC-NBS-LRR family of resistance proteins and requires the signaling gene *NDR1* for full activity. Recognition of ATR39-1 in Wei-0 does not inhibit growth of *Hpa* strains expressing the effector, suggesting complex mechanisms of pathogen evasion of recognition, and is similar to what has been shown in several other cases of plant-oomycete interactions. Identification of this resistance gene/effector pair adds to our knowledge of plant resistance mechanisms and provides the basis for further functional analyses.

## Author Summary

Oomycete plant pathogens are among the most devastating agricultural pests and employ arsenals of effector proteins to manipulate their plant hosts. Some of these effectors, however, are recognized in the plant and trigger an immune response. *Hyaloperonospora arabidopsidis (Hpa)* causes downy mildew on the model plant *Arabidopsis thaliana* and this interaction has been developed as a model system for oomycete pathogenesis. Here, we employ computational predictions to identify a novel effector ATR39-1, which is highly conserved among different *Hpa* isolates. A two amino acid-insertion in the alternative allele ATR39-2 correlated with evasion of recognition. We identified the corresponding resistance gene *RPP39* and found that the signaling gene *NDR1* is required to establish full resistance. Recognition of ATR39-1 by RPP39 in the plant did not inhibit growth of the oomycete, suggesting that complex mechanisms exist to prevent effector recognition. Knowledge of such novel resistance interactions provides the backbone of our understanding of plant resistance mechanisms and will aid in the further dissection of plant immunity.

## Introduction

Oomycetes comprise a number of agriculturally important plant pathogens, including *Phytophthora infestans* (potato and tomato late blight), *P. ramorum* (sudden oak death) and *P. sojae* (soybean root rot). *Hyaloperonospora arabidopsidis* (*Hpa*, downy mildew, formerly known as *Peronospora parasitica*) is a naturally occurring oomycete pathogen of the model plant *Arabidopsis thaliana*. The *Hpa*/*Arabidopsis* pathosystem allows the scientific community to take advantage of the genetic tools developed for *Arabidopsis* in the dissection of plant responses to oomycetes [1]. During their parasitic life stages, oomycetes deliver an arsenal of effector proteins to their plant host, which are hypothesized to target basal defense mechanisms and/or manipulate host metabolism to extract nutrients for the pathogen [2]. The first oomycete effector proteins were, however, identified based on their avirulence functions, i.e. their presence triggered an immune response in the host resulting in resistance to the pathogen. This so-called effector triggered immunity (ETI) is characterized by the specific recognition of pathogen avirulence effectors by plant resistance receptors, either directly or indirectly [3]. ETI is often accompanied by localized cell death at the site of infection, the hypersensitive response (HR), which limits the spread of biotrophic pathogens inside the plant host [4]. The *Hpa* effectors *Arabidopsis thaliana* recognized 1 (ATR1), ATR13 and ATR5 as well as Avr1b from *P. sojae* were identified using classic genetic crosses between virulent and avirulent pathogen strains [5]–[8], and have been shown to be under strong positive selection. *P. infestans* effector Avr3a, on the other hand, was isolated using association genetics [9]. Interestingly, despite having no sequence homology, Avr3a and ATR1 reside in conserved syntenic regions within the genome [9] and their three-dimensional structures reveal a similar fold between Avr3a and a sub-domain of ATR1 [10]–[13].

All currently described oomycete effectors were found to have a modular domain structure, containing amino-terminal domains involved in effector translocation and carboxyl-terminal effector domains. The translocation domains typically include a secretion signal sequence followed by the amino acid motif Arg-x-Leu-Arg (RxLR), in which x could be any amino acid. The RxLR motif was shown to be important in translocation of effectors to the host cytoplasm and it is also functionally interchangeable with the translocation motif of *Plasmodium falciparum* effectors [14], [15]. The absence of a canonical RxLR motif in the recently cloned effector ATR5 suggests that other sequences may also be involved in translocation of effectors [8]. The carboxyl-terminal effector domains are highly divergent and typically do not have strong sequence similarity to other proteins.

The genomes of several *Phytophthora* species as well as of *Hpa* strain Emoy2 have recently been sequenced and this has fueled bioinformatics efforts to elucidate the complete arsenal of effectors [16]–[18]. Effector predictions were based on the presence of the amino-terminal translocation domains. While bacterial pathogens such as *Pseudomonas syringae* contain around 30 to 40 effector genes [19], the oomycete genomes were found to contain expanded effector repertoires, ranging from around 350 in *P. sojae* and *P. ramorum* to more than 700 in *P. infestans* [20]. Initial effector predictions from the *Hpa* genome yielded 149 RxLR effector genes [21], however, the published genome sequence for *Hpa* strain Emoy2 contains only 134 annotated RxLR effectors [17]. Currently, major efforts are being undertaken in dissecting the effector complement of several oomycetes in order to identify novel avirulence determinants as well as to define effector virulence functions. Several recent large-scale effector screens in different oomycetes focused on the localization of effectors in the plant cell during infection and on their roles in facilitating oomycete infections [22], [23]. These transgenic approaches identified effectors that localize to the oomycete haustorial feeding structures and may be important in mediating intercellular communication. Another study employed mining of expressed sequence tags (ESTs) to identify genes highly expressed during *Hpa* infection, and investigated their potential functions during compatible interactions [24]. An *in planta* expression screen of *P. infestans* effectors was successful in identifying the cognate avirulent effectors ipiO/AVRblb1 and AVRblb2, recognized by the R proteins Rpi-blb1 and Rpi-blb2, respectively [25], [26].

Resistance to different strains of *Hpa* was mapped to a number of *RPP* (*Resistance to Peronospora parasitica*) loci in several *Arabidopsis* ecotypes [27], [28]. Six of these *R* genes were subsequently cloned, but the corresponding recognized effectors have only been identified for three of them, RPP1, RPP13 and recently RPP5, which recognize ATR1, ATR13 and ATR5 respectively [5], [7], [8]. Similar to the *R* genes that function against other microbial pathogens, *RPP* genes belong to the large Nucleotide Binding Site-Leucine Rich Repeat (NBS-LRR) gene family in *Arabidopsis*, which comprises a total of around 150 members, but only a few with an assigned function [29]. Characterized *R* genes confer resistance to various classes of pathogens including oomycetes, bacteria, fungi and viruses. Additionally, NBS-LRR genes have been implicated in non-self recognition in inter-accession hybrids [30].

Research on RPP1/ATR1 and RPP13/ATR13 has greatly advanced our understanding of effector recognition and resistance signaling. Recognized ATR1 alleles have been shown to associate *in planta* with the LRR-domain of RPP1 before triggering an immune response [31]. Intracellular recognition of ATR13 by the CC-NBS-LRR protein RPP13 was shown to signal independently of the known signaling genes *EDS1* and *NDR1*, indicating the presence of additional signaling pathways activated upon effector recognition [32].

In order to gain a better understanding of the interactions between *Hpa* and its host *A. thaliana*, we set out to screen 83 *Arabidopsis* ecotypes for novel recognition specificities with a subset of predicted *Hpa* effectors. Here, we describe a successful approach to mine the *Hpa* genome for functional effector proteins based on domain structure similarity to known oomycete RxLR effectors. We identified a novel avirulent RxLR effector, ATR39, which is recognized by the *Arabidopsis* ecotype Weiningen. Comparison of ATR39 alleles identified two amino acids that are critical for recognition. We cloned the corresponding *R* gene, *RPP39*, and showed that it is a member of a small cluster of CC-NBS-LRR genes and requires *NDR1* for downstream signaling of plant defense responses. Our ability to combine computational predictions with molecular and genetic techniques will facilitate the rapid identification of novel *R* genes as well as inform our understanding of the evolution of pathogenesis and resistance.

## Results

### HMM prediction of effectors from the *Hpa* genome sequence

**Figure 1:**
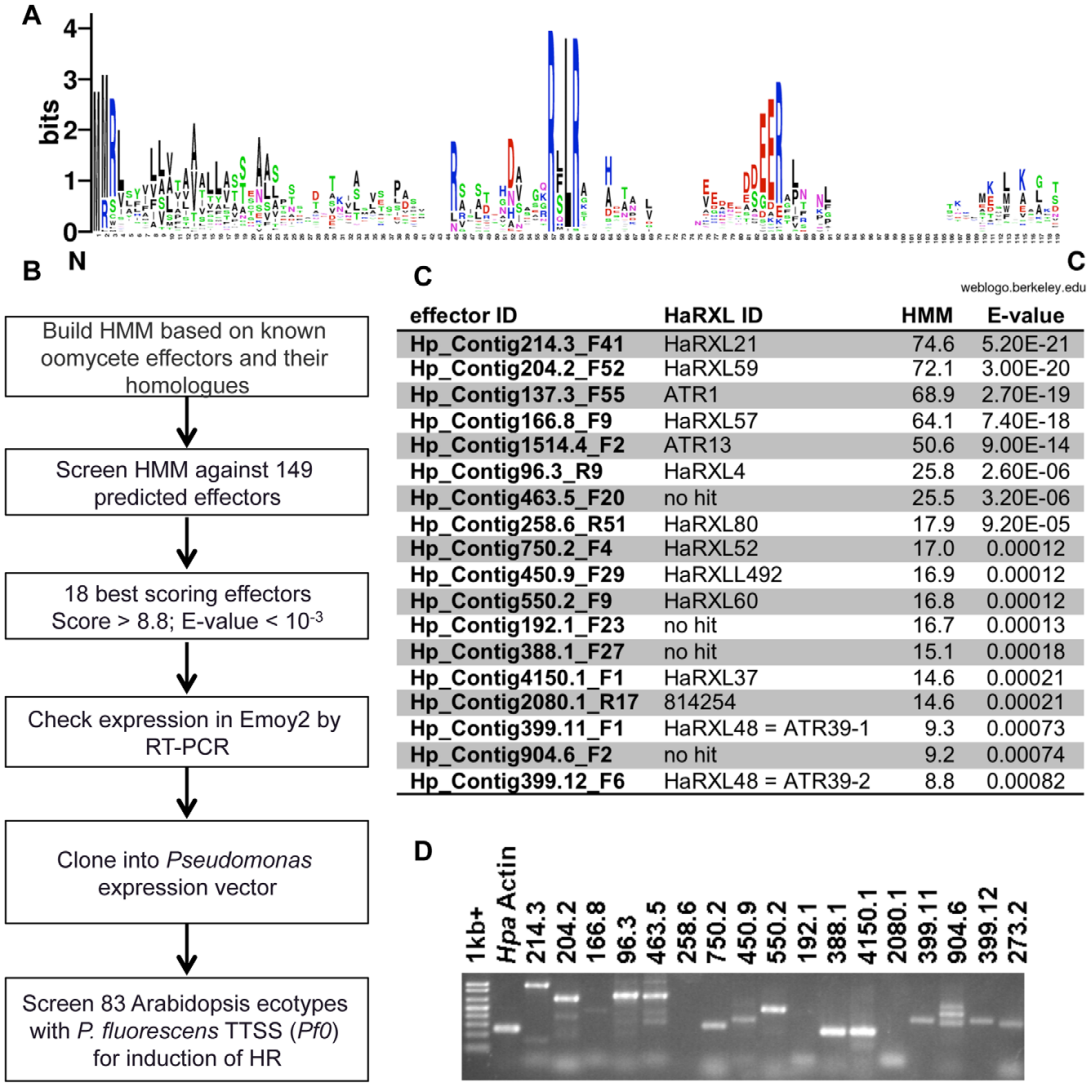
Identification of functional avirulence effector candidates from *Hyaloperonospora arabidopsidis (Hpa)* using a Hidden Markov Model (HMM)–based search. A) Consensus sequence of 43 oomycete effectors used in building the HMM. The consensus was generated using the Weblogo program. Larger letters signify higher conservation. B) Screening scheme for identification of effectors. The steps in the pipeline are as follows: 1) Building HMM model based on N-terminal secretion signal and RXLR domains of known effectors; 2) Screening the HMM against 149 predicted effectors to form a prioritized list; 3) Focusing on 18 best scoring effectors with HMM score >8.5 and E-value <0.001; 4) Determining expression of predicted effectors in *Hpa* Emoy2 7 days post-infection (dpi); 5) Cloning expressed effectors in *Pseudomonas* vector pPsSP [35] or pEDV3 [36] and determining expression in *P. fluorescens*; 6) Screening 83 *Arabidopsis* ecotypes with effectors for induction of resistance response, visible as HR. C) Summary of highest scoring effector candidates identified by HMM search. Effector IDs correspond to position on contigs in *Hpa* genome version 7.0. HaRXL IDs are the annotated IDs in the published *Hpa* genome version 8.3. “No hit” indicates genes not annotated in the final genome. HMM scores and E-values are indicated. D) Expression of effectors in *Hpa* Emoy2 infected tissue 7 dpi as determined by RT-PCR.

Based on characterized effectors from *Phytophthora* sp. and *Hpa*, intracellular oomycete effector proteins are predicted to contain several conserved domains: an N-terminal secretion signal peptide (SP), a central RxLR motif, and a C-terminal variable effector domain. Previously, Win et al. mined the *Hpa* genome (version 7.0) for predicted open reading frames of >70 amino acids, which contained the N-terminal SP and the RxLR motif between amino acids 30 and 60, and found 149 effectors fulfilling these criteria [21]. In order to refine this search we generated a Hidden Markov Model (HMM) from the N-terminal conserved domains (SP and RxLR) of previously identified effectors and their homologues (this set includes 43 proteins, [21], Figure 1A). HMMs are widely used to predict homologies with statistical significance, most prominently in the protein domain database Pfam [33]. This method allowed us to screen the 149 initially predicted effectors with the HMM model and prioritize them for experimental validation, as outlined in Figure 1B. We decided to focus downstream characterization on the 18 highest-scoring predicted effectors, with E-value scores <0.001 (Figure 1C). Amino acid and nucleotide sequences for these effectors are available in fasta format online as File S1 and S2, respectively. Interestingly, some of the predicted effectors scored even higher than two known effectors, ATR1 (Hp_Contig137.3_F55) and ATR13 (Hp_Contig1514.4_F2). We tested whether the predicted effectors were expressed during *Hpa* infection by RT-PCR and confirmed expression for 15 of them at seven days post-inoculation (Figure 1D).

### Generating a system to screen for novel recognition specificities

Because of *Hpa*'s obligate biotroph lifestyle, studies on *Hpa* have relied on surrogate systems, delivering oomycete effectors biolistically [34] or using bacterial or viral vectors [35], [36].

**Figure 2:**
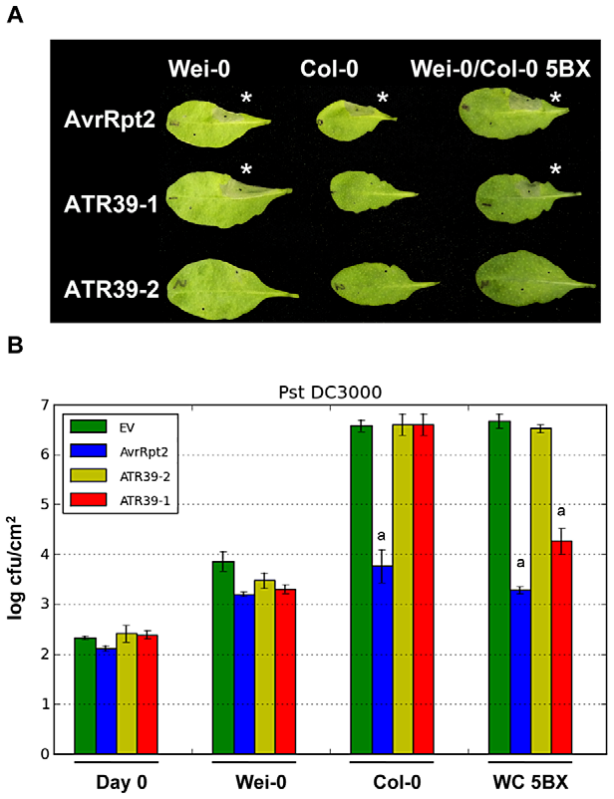
*Hpa* effector ATR39-1 is recognized by *A. thaliana* ecotype Wei-0. A) ATR39-1, but not ATR39-2, triggers a hypersensitive response (HR) in the *Arabidopsis* ecotype Weiningen (Wei-0) when delivered by *Pf*0. The top half of the leaf was inoculated with the indicated effector, the bottom half with a negative GFP control. Wei-0 also contains a functional allele of *RPS2* and *AvrRpt2* was included as a positive control for HR. The picture was taken 24 hours post infection. Stars highlight induction of HR. B) ATR39-1 recognition inhibits growth of virulent *Pst* DC3000 in a Col-0 line where the *RPP39* locus was introgressed (WC 5BX), while Wei-0 displays natural resistance towards *Pst* DC3000. Plants were inoculated with *Pst* DC3000 expressing the indicated effectors (10^5^ cfu/mL) and bacterial titers determined 0 and 4 days post-infection. Error bars indicate standard deviation. Significant differences as determined by Student's t-test are indicated by small letters (P<0.0003). Experiments were repeated at least twice with similar results.

We PCR amplified the highest scoring expressed effectors from the Emoy2 isolate of *Hpa*, past the predicted signal peptide cleavage site, and cloned them into two *Pseudomonas* expression vectors. First, the effectors were shuttled into a Gateway-compatible *Pseudomonas* vector as C-terminal fusions with the AvrRpm1 type three secretion system (TTSS) signal peptide (pPsSP, [35]). However, we observed that ATR1 was not functional in this system, but was functional as a C-terminal fusion with the AvrRps4 TTSS signal peptide in the alternative pEDV3 system [36]. We thus decided to also subclone the predicted effectors into pEDV3 and test them in both expression systems. We conjugated these expression plasmids into *Pseudomonas fluorescens (Pf0)*, a non-pathogenic *Pseudomonas* strain that lacks an endogenous TTSS and effectors and was engineered to express the *Pseudomonas syringae* pv. tomato (*Pst*) DC3000 *hrp* cluster and TTSS [37]. Using this strain allowed us to deliver individual effectors to the plant host, circumventing considerable background we often observed when inoculating a variety of ecotypes with *Pst* DC3000 (data not shown). We did not observe a reaction to *Pf0* carrying an empty vector control in most ecotypes, even after 48 hours post-inoculation (hpi). Effector recognition on the other hand resulted in a visible hypersensitive response within 24 hpi (Figure 2A). We then screened 83 *Arabidopsis* ecotypes from the Nordborg collection (1, [38]) with *Pf0* expressing each of the predicted effectors.

### *Hpa* effector ATR39-1 is recognized by *Arabidopsis* ecotype Weiningen

One of the predicted effectors, Hp_Contig399.11_F1, triggered a visible HR in the ecotype Weiningen (Wei-0), when delivered by *Pf0* (Figure 2A). Hp_Contig399.11_F1 has an HMM score of 9.3 and ranks number 16 of the predicted effectors (Figure 1C). None of the other 82 available ecotypes from the Nordborg collection displayed an HR upon delivery of Hp_Contig399.11_F1. According to nomenclature previously applied to *Hpa* effectors, we renamed this effector ATR39-1 (for *A. thaliana* recognized 39-1, accession number JQ045572).

In order to assess whether ATR39-1 conferred avirulence to pathogenic bacteria, we conducted bacterial growth assays but found that Wei-0 showed natural resistance towards several pathogenic *Pseudomonas* strains, including *Pst* DC3000 (Figure 2B) and *P. syringae* pv. *maculicola (Psm)* ES4326 (Figure S1). We therefore generated an introgression line in which the Wei-0 recognition locus was introduced into the Col-0 background by repeated backcrossing. Using this line (WC-5BX), we showed that ATR39-1 is indeed recognized by the Wei-0 locus and that this recognition results in decreased growth of *Pst* DC3000 (Figure 2B).

Unlike ATR1, ATR39-1 does not contain the acidic DEER (Asp-Glu-Glu-Arg) motif following the RxLR translocation motif. We also tested whether the effector-truncation past the RxLR motif triggers the hypersensitive response and found that the effector domain (ATR39-1 **Δ**48) is also able to trigger an HR when delivered by *Pf0* (Figure S2).

### ATR39 has two polymorphic alleles that are highly conserved among *Hpa* isolates

**Figure 3:**
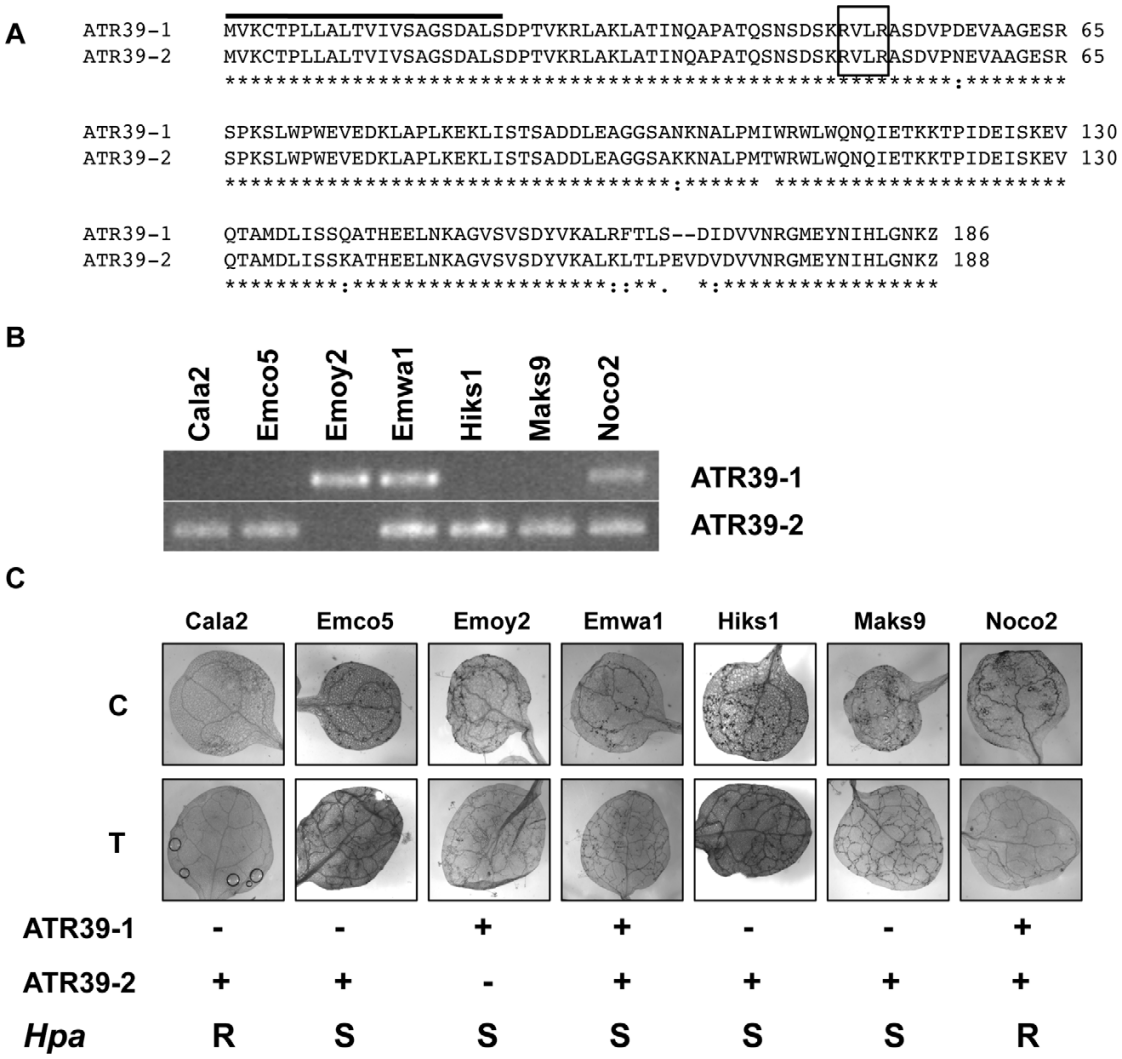
*ATR39* alleles are expressed in different *Hpa* isolates, but expression of *ATR39-1* does not correlate with avirulence on Wei-0. A) Amino acid sequence alignment of ATR39-1 and ATR39-2 using ClustalW. B) RT-PCR with allele-specific primers from plants infected with the indicated *Hpa* isolates, harvested at 7 dpi. Sequencing of the amplified *ATR39* fragments revealed the absence of additional polymorphisms in *Hpa* strains. C) Trypan blue staining of Wei-0 leaves inoculated with the indicated *Hpa* strains. Presence or absence of *ATR39* alleles is indicated below, as well as qualitative scoring of *Hpa* growth on Wei-0. C, cotyledons; T, true leaves; S, susceptible; R, resistant.

Using the same primers we amplified two alleles of ATR39 from *Hpa* strain Emoy2, but only one of them, ATR39-1, is recognized by Wei-0 (Figure 2). Both alleles are predicted in our HMM search with scores of 9.3 and 8.8 for ATR39-1 and ATR39-2, respectively. The two alleles differ by 10 nucleotides, resulting in 9 amino acid substitutions, and a 2 amino acid insertion in ATR39-2 relative to ATR39-1 (Figure 3A). In the published *Hpa* Emoy2 genome assembly ATR39-1 is not annotated, however, its allelic variant ATR39-2 is annotated as HaRxL48 [17].

We have generated Illumina paired-end sequencing data for the Emwa1 isolate of *Hpa* and could identify both alleles in the assembly (data not shown). In this assembly we do not see signatures of duplication events, suggesting that *Hpa* Emwa1 and possibly also Emoy2 and Noco2 are heterozygous at this locus.

Using allele-specific primers we amplified ATR39 alleles from seven isolates of *Hpa* and found that *ATR39-1* is only present and expressed in Emoy2, Emwa1 and Noco2 isolates (Figure 3B). ATR39-2, on the other hand, is more prevalent in this set of *Hpa* isolates. Surprisingly, we could not amplify ATR39-2 from the Emoy2 isolate currently grown in our lab. Since we initially amplified the effectors from a DNA sample obtained from a different source than the *Hpa* Emoy2 strain, and there is evidence for heterozygosity in the various lab strains, it is possible that our current lab strain is now homozygous for ATR39-1. In order to determine whether this strain is Emoy2, we amplified and sequenced the ATR1 effector and verified that our lab strain is indeed Emoy2 (data not shown).

We sequenced the ATR39 amplification products and found no nucleotide polymorphisms among ATR39-1 or ATR39-2 alleles from the different *Hpa* isolates. This conservation is in stark contrast with other characterized *Hpa* effectors ATR1, ATR5 and ATR13, which are highly divergent [5], [8], [39]. These findings indicate that the two ATR39 alleles may have an important function in *Hpa* and may be maintained under strong balancing selection. Sequence comparisons and pattern searches yielded no obvious homologs or putative function for ATR39. Taken together, we have identified a novel effector from *Hpa* with unknown function that is able to trigger a resistance response in *Arabidopsis*.

### Recognition of ATR39-1 is suppressed during *Hpa* infection

The Wei-0 ecotype was previously shown to be susceptible to multiple *Hpa* isolates [40]. We confirmed that despite being able to recognize ATR39-1 present in Emoy2, Emwa1 and Noco2, Wei-0 still supports growth of these isolates (Figure 3C). This lack of resistance is not due to the lack of *ATR39-1* transcript since we detected *ATR39-1* expression in infected tissue using RT-PCR (Figure 3B and Figure S3). A similar suppressed recognition phenotype was observed for one of the ATR1 alleles. The ATR1^Emco5^ allele is recognized in several *Arabidopsis* ecotypes, which remain susceptible to infection by *Hpa* Emco5 [5], [41].

### Recognition of ATR39 is abolished by E168/V169 in ATR39-2

**Figure 4:**
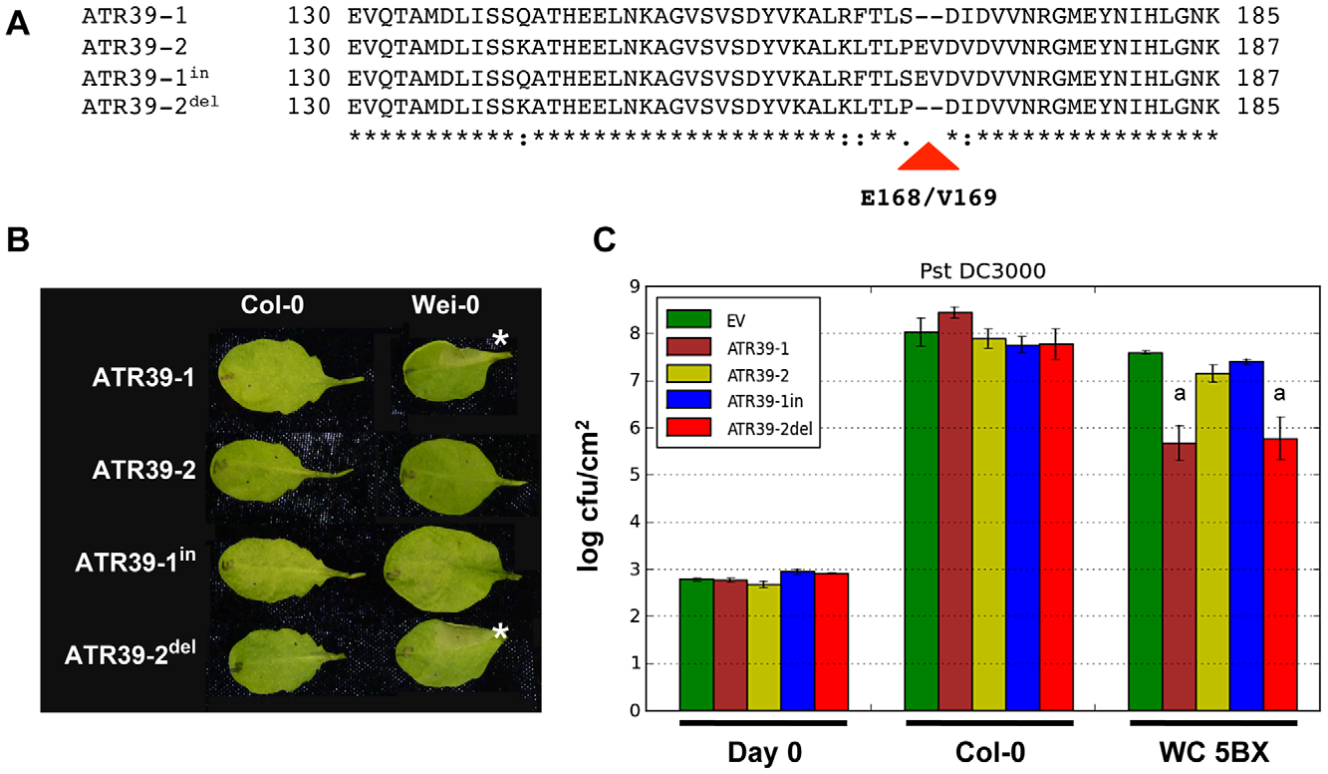
Amino acids E168/V169 abolish recognition of ATR39. A) Alignment of the C-terminal regions of ATR39 alleles and their corresponding insertion/deletion constructs. E168/V169 are indicated by a triangle. B) Only alleles lacking E168/V169 trigger an HR when delivered by *Pf*0. The lower half of each leaf was inoculated with an empty vector control. Pictures were taken 24 hpi. Stars highlight positive HR. C) *Pst* DC3000 growth assay with indicated ATR39 insertion/deletion alleles. Significant differences as determined by Student's t-test are indicated by small letters (P<0.005). Experiments were repeated at least twice with similar results.

ATR39-1 and ATR39-2 differ by 9 non-synonimous substitutions, and a two amino acid insertion in ATR39-2. In order to define the region in ATR39 responsible for differential recognition of the two alleles, we generated ATR39-1^in^ by inserting E168/V169 into ATR39-1 using site-directed mutagenesis. Similarly, we generated ATR39-2^del^, in which E168/V169 were deleted (Figure 4A). ATR39-2^del^, but not ATR39-1^in^ triggered an HR in *Arabidopsis* Wei-0 suggesting that the presence of amino acids E168/V169 blocks recognition of ATR39 (Figure 4B). Additionally, we performed *Pst* DC3000 growth assays and found that ATR39-2^del^ restricted bacterial growth, whereas *Pst* DC3000 expressing ATR39-1^in^ grew to similar levels as the non-recognized allele ATR39-2 or empty vector control (Figure 4C). These findings suggest that amino acids E168/V169 in ATR39-2 are critical in evading recognition by the cognate R protein.

### *RPP39* is a member of a CC-NBS-LRR gene cluster

**Figure 5:**
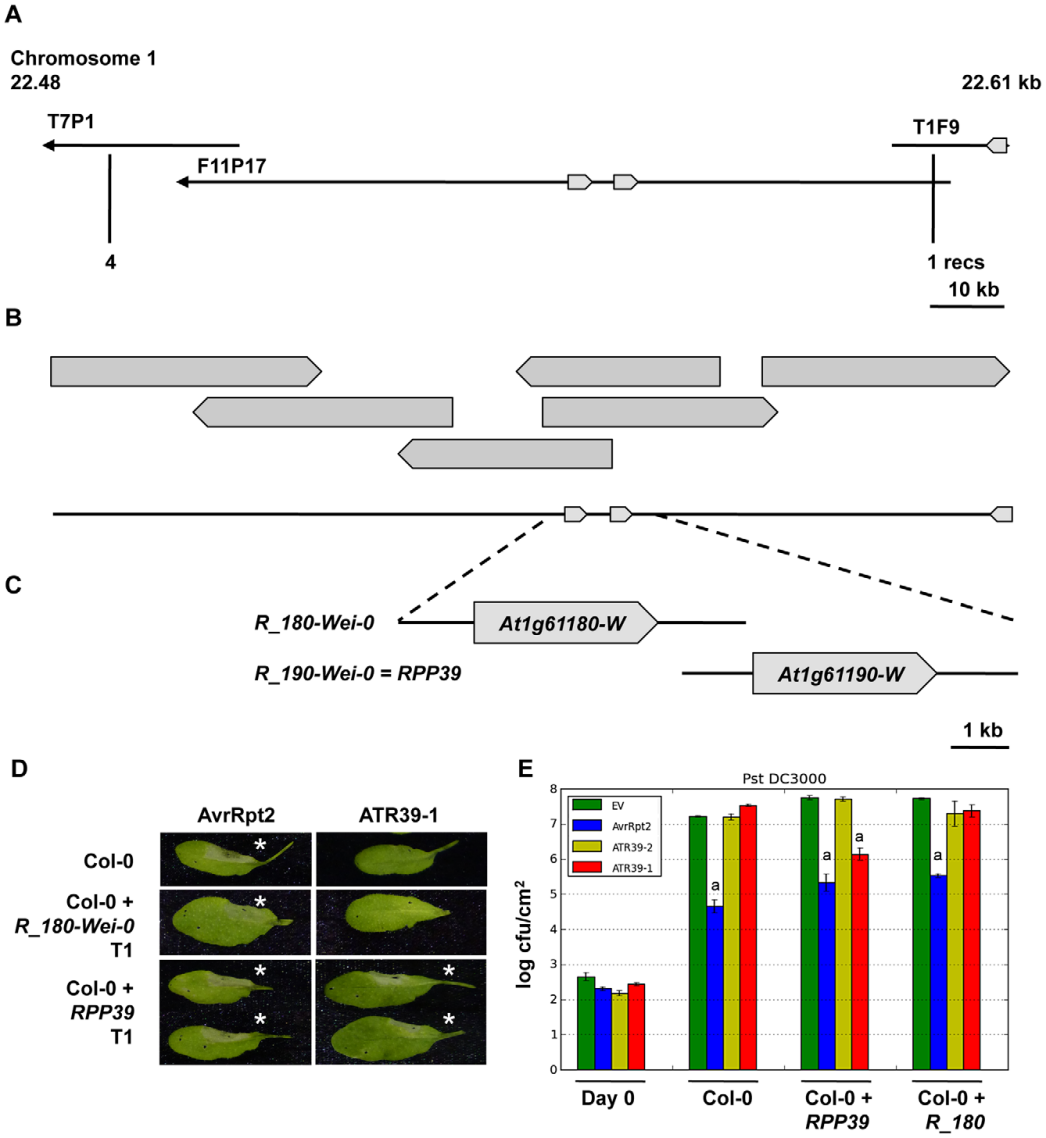
Map-based cloning of *RPP39*. A) Map location of *RPP39* on Col-0 BAC clones of Chromosome 1. Predicted NBS-LRR *R* genes are indicated as block arrows. Positions of the last flanking markers and numbers of recombinants are indicated below. B) Wei-0 fosmid contig spanning the 133 kb locus containing *RPP39*. C) Genomic clones of the *R* genes at the *RPP39* locus used in functional analysis in transgenic Col-0 *Arabidopsis*. D) HR induced by ATR39-1 and AvrRpt2 in transgenic *Arabidopsis* Col-0 T1 transformed with the *R* gene candidates in C. The lower half of each leaf was inoculated with an empty vector control. Pictures were taken 24 hpi. Stars highlight positive HR. E) *Pst* DC3000 growth assay on select T2 transgenics containing *R* gene candidates. Significant differences as determined by Student's t-test are indicated by small letters (P<0.0003). Experiments were repeated at least twice with similar results.

Resistance to *Hpa* strains is mediated by a number of *RPP* (*Resistance to Peronospora parasitica*) loci. In order to identify the *RPP39* gene responsible for recognition of ATR39-1 we generated a cross between Wei-0 and Col-0 and found that recognition segregated as a single dominant locus in the F2 progeny. Using 886 F2 plants, we delineated the *RPP39* locus to a 150 kilobase region on the bottom of chromosome 1. In Col-0 this region contains two homologous CC-NB-LRR genes with 91% identity arranged in a tandem repeat (*At1g61180* and *At1g61190*). Because there is no sequence information available for Wei-0, we generated a fosmid library and identified six overlapping clones that span this region (Figure 5B). We sequenced the fosmids using Illumina next generation sequencing and found several rearrangements and a transposon insertion relative to the Col-0 sequence (Figure S4). Wei-0 also contains two CC-NBS-LRR genes at this locus, which were the most promising candidates for *RPP39*.

We amplified genomic regions containing the two candidate *R* genes, *R_180-Wei-0* (accession number JQ045574) and *R_190-Wei-0* (accession number JQ045573) and transformed them into *Arabidopsis* Col-0 for complementation. Only transgenic plants containing the gene corresponding to the *At1g61190* locus (*R_190-Wei-0*) developed an HR in response to ATR39-1 delivery, indicating that this gene is *RPP39* (Figure 5D). Growth assays with *Pst* DC3000 expressing ATR39-1 performed on plants in the T2 generation confirmed these results as we observed reduction in bacterial growth only in plants containing the *R_190-Wei-0*/*RPP39* transgene (Figure 5E).

**Figure 6:**
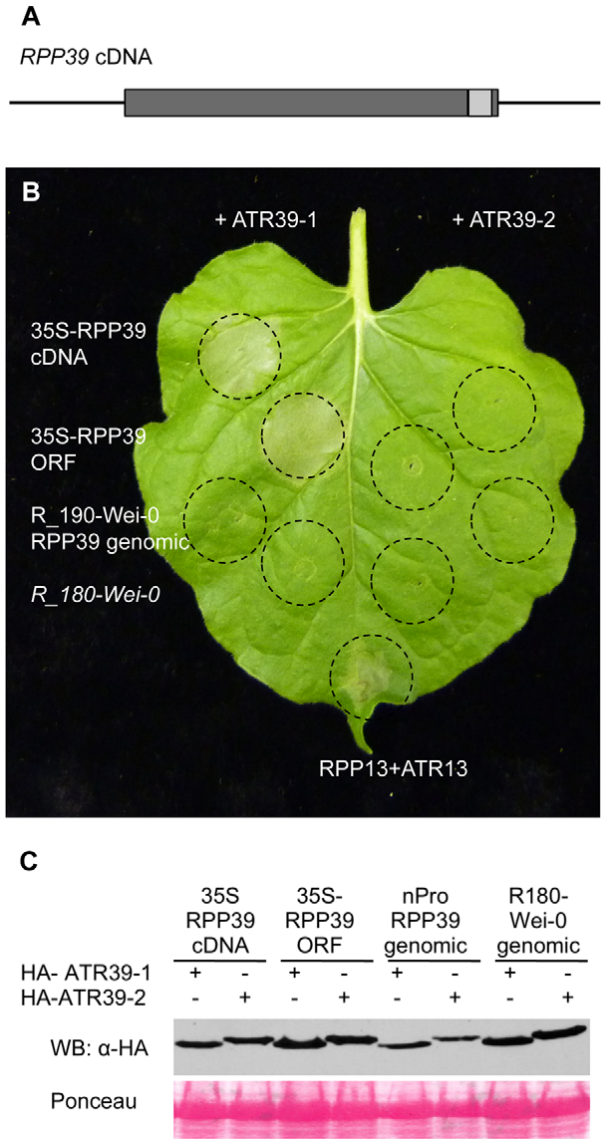
*RPP39* cDNA triggers ATR39-1–dependent HR in transient expression assays in *Nicotiana benthamiana*. A) Gene structure of *RPP39*. Promoter and terminator regions are indicated as black lines, exons are shown as dark grey boxes and the intron as a light grey box. B) *Agrobacterium*-mediated transient expression assay in *N. benthamiana*. The indicated *RPP39* clones and *R_180-Wei-0* were inoculated together with HA-ATR39-1 (left) and HA-ATR39-2 (right) at OD_600 nm_ = 0.5 each. Inoculated areas are delineated by dashed lines. The picture was taken 72 hpi. RPP13 co-inoculated with ATR13^Emco5^ was included as positive control. C) Protein immunoblot of HA-ATR39 showing similar levels of protein expression. Samples were collected 24 hpi and probed with α-HA antibody. Ponceau staining is included to show equal loading of the samples.

The predicted *RPP39* coding region contains an intron close to the C-terminus, connecting a large N-terminal exon with a short C-terminal exon encoding the last 15 amino acids (Figure 6A). We amplified *RPP39* from Wei-0 cDNA and confirmed the presence of this intron. In *Agrobacterium*-mediated transient expression experiments in *Nicotiana benthamiana* we showed that the *RPP39* cDNA driven by the CaMV35S promoter is able to trigger ATR39-1 dependent HR (Figure 6B). Interestingly, expression of the genomic *RPP39* clone in *N. benthamiana* was not sufficient to trigger HR, yet it was functional in transgenic *Arabidopsis*. Because a 35S driven clone of the genomic sequence of *RPP39* is able to trigger HR (Figure 6B), we believe that the lack of responsiveness of the genomic *RPP39* clone is probably due to low expression off the native promoter in the transient assay.

RPP39 is very similar to its Col-0 paralogs, At1g61190 (NM_104800.1) and At1g61180 (NM_104799.3), with 86% identity at the nucleotide level and 81% to 87% at the amino acid level (Figure S5). The homologs are most divergent in the C-terminal LRR domain (Figure S6). The most closely related R proteins outside the RPP39 cluster are RPS5 (NP_172686.1), RFL1 (AAL65608.1) and RPS2 (AAA21874.1) with 50%, 49% and 27% identity to RPP39 at the amino acid level, respectively (Figure S5). Taken together, we identified a functional resistance gene as a member of small *R* gene cluster.

### RPP39 requires NDR1 for full resistance

**Figure 7:**
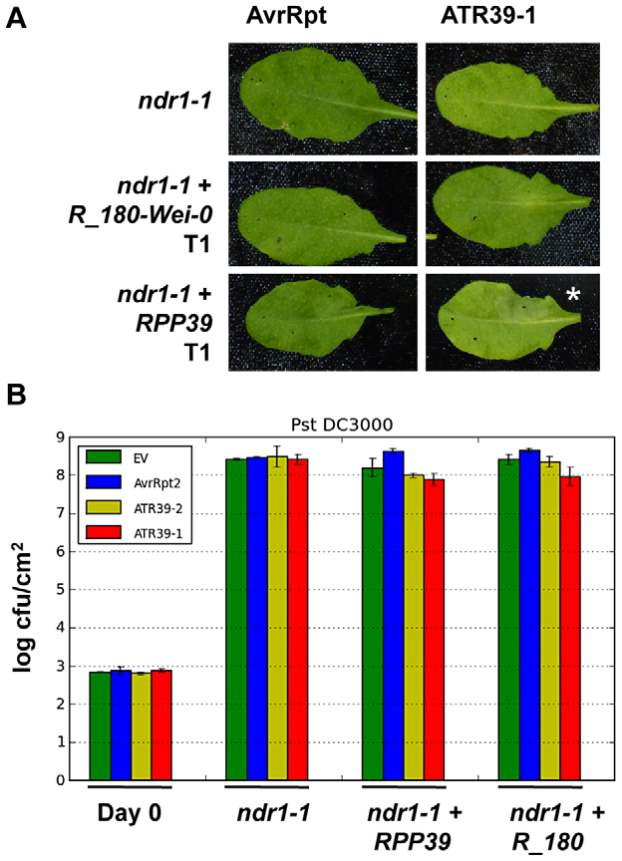
NDR1 is required for full resistance but dispensable for HR mediated by RPP39. A) *Pf*0 inoculation of T1 transgenic *Arabidopsis ndr1* mutant plants transformed with the indicated *R* genes. The lower half of each leaf was inoculated with an empty vector control. Pictures were taken 24 hpi. Stars highlight positive HR. B) *Pst* DC3000 growth assay on select T2 transgenic *ndr1* plants transformed with the indicated *R* gene candidates. Statistical analysis using Student's t-test identified no significant differences. Experiments were repeated at least twice with similar results.

*Non-specific disease resistance 1 (NDR1)* is a common signaling gene required for CC-NBS-LRR mediated resistance functions [42]. Since *RPP39* is similar to the resistance genes *RPS2* and *RPS5*, both of which require *NDR1*, we investigated the involvement of *NDR1* in *RPP39* mediated resistance. We generated stable transgenic plants containing *RPP39* or its non-functional paralog *R180_Wei-0* in the *ndr1* mutant background. *RPP39* transgenic plants displayed no difference in their ability to trigger ATR39-1-dependent HR as compared to transgenics in Col-0 wild type background (Figure 7A). However, in *Pst* DC3000 growth assays we did not see a similar growth reduction in the *ndr1* transgenics (Figure 7B). These results indicate that the ability of RPP39 to trigger HR is separable from complete disease resistance, and is reminiscent of RPM1, which displays similar NDR1 dependency [43].

## Discussion

We have used a combination of computational prediction methods and phenotypic screening to identify a novel recognized effector from *Hpa*. Being an obligate biotroph, *Hpa* is currently not amenable to in depth genetic analysis, and cloning of the previously identified RxLR effectors ATR1 and ATR13 was a lengthy and cumbersome process [5], [7]. In our approach, we mined the *Hpa* genome for putative effector sequences and ranked them in a comparison with a Hidden Markov Model (HMM) generated based on the N-terminal conserved translocation domains of previously identified oomycete effectors. When we generated the HMM, all characterized effectors contained an RxLR and in our initial ranking we screened the HMM only against predicted RxLR effectors. Recently, Bailey et al. cloned and characterized ATR5 from *Hpa* strain Emoy2, the first recognized effector without a canonical RxLR sequence, suggesting that this motif can be modified [8]. In accordance with this, when screening the HMM against all annotated proteins in the final *Hpa* genome release [17], we also identified several non-RxLR variants within the top-scoring effector candidates, including ATR5 (data not shown). These findings suggest that our screen is by no means exhaustive and that more recognized effectors await characterization. When screening the HMM against the published annotation of the *Hpa* protein database, we also noticed that a few of our predicted effectors did not appear in the results because their annotated genes do not include the N-terminal translocation domains due to a difference in annotation of the translational start site.

Our choice of using the non-pathogenic *P. fluorescens* that does not normally trigger a response in *Arabidopsis* as a surrogate expression and delivery system allowed us to rapidly screen a large number of *Arabidopsis* ecotypes for novel recognition specificities. Interestingly, despite the fact that *Hpa* strains are predicted to contain between 10 and 20 avirulent effectors, according to association studies in the 1990s [44], in our set we only identified one novel effector that was able to trigger resistance in an *Arabidopsis* ecotype. Under our assay conditions and using our expression vectors we did not detect consistent phenotypes (either virulence or avirulence) for most of the tested effectors from the Emoy2 isolate of *Hpa*. Notably, in this study we compared the two delivery systems pEDV3 and pPsSP, which fuse the TTSS signal peptides from the bacterial effectors AvrRps4 and AvrRpm1, respectively, upstream of the *Hpa* effector [35], [36]. Intriguingly, ATR39-1 was functional when expressed as AvrRpm1 fusion in the pPsSP vector, but not as AvrRps4 fusion in the pEDV3 vector. ATR1, on the other hand, did not trigger responses when delivered as AvrRpm1 fusion protein in the pPsSP system and is only functional in the pEDV3 system. These results suggest that the choice of expression system can greatly influence the observed phenotypes and should be taken into account. Therefore, we cannot dismiss the possibility that several of the predicted effectors might be functional in different assay conditions or recognized by different *Arabidopsis* ecotypes not tested in this study. In a set of *Arabidopsis* ecotypes from the United Kingdom, where all currently available *Hpa* strains originate from, several ATR13 alleles were tested and were found to be recognized by RPP13 alleles in different ecotypes [45]. The fact that we did not observe prevalence of effector recognition in our subset of *Arabidopsis* ecotypes suggests that the co-evolution between pathogen and host may play an important role in this obligate biotroph interaction. We also screened various alleles of ATR1 and ATR13 on the Nordborg collection of *Arabidopsis* ecotypes [38] and found six ecotypes capable of recognizing ATR1^Emoy2^, including Ws-0 and Nd-1, but none that recognized ATR13^Emoy2^ [41]. Compared with the prevalence of recognition specificities for bacterial effectors such as AvrRpt2 or AvrPphB, which are recognized by RPS2 and RPS5 in more than half of the tested ecotypes [38], these results indicate a more dynamic effector repertoire in *Hpa*.

We identified two amino acids, E168 and V169, which abrogate recognition of ATR39-2. Deletion of these amino acids in ATR39-2 resulted in a gain-of-recognition phenotype while insertion of E168/V169 in ATR39-1 lead to loss-of-recognition by RPP39. It is possible that this insertion/deletion polymorphism alters the three dimensional structure of ATR39, thus disrupting the interaction with potential target proteins. Another possibility would be that the polymorphism alters putative enzymatic properties of ATR39. However, since the primary amino acid sequence of ATR39 is not homologous to any known protein, we can only speculate about the consequences of the insertion on the function of this protein. Experiments to determine the structure and to identify interacting proteins of ATR39 alleles will help elucidate the function of this novel effector.

The *Arabidopsis* ecotype Wei-0 exhibits an interesting resistance pattern: it is resistant against several tested bacterial pathogens but highly susceptible towards *Hpa* isolates ([40], this study). Intriguingly, despite being able to recognize ATR39-1 when delivered by a surrogate system, Wei-0 is not able to restrict growth of *Hpa* expressing ATR39-1. This finding is reminiscent of ATR1^Emco5^, which is recognized in the *Arabidopsis* ecotype Ws-0 by RPP1-WsB, but this recognition does not abrogate growth of *Hpa* in a natural infection. Additional *Arabidopsis* ecotypes recognizing ATR1^Emco5^ were recently identified, and these were also shown to support growth of *Hpa* isolate Emco5, indicating similar mechanisms in virulence may act in these interactions [41]. These data suggest that *Hpa* may contain a pathogenicity factor, perhaps another secreted effector, which prevents recognition of ATR1 in Emco5 or ATR39-1 in Emwa1 and Emoy2. Support for this hypothesis comes from a study on different alleles of the *P. infestans* effector ipiO/Avrblb1, where expression of one allele, ipiO4, was found to suppress resistance mediated by the ipiO1/Rblb1 interaction [46]. Alternative explanations for the lack of recognition in these instances could be inhibition of effector translocation to the host, mistimed expression of either effector or *R* gene or incomplete resistance mediated by the *R* gene that is not strong enough to contain the pathogen. Experiments aimed at investigating this interesting phenotype should yield important insight into *Hpa* virulence.

*RPP39* is a member of a small cluster of CC-NBS-LRR genes, which is rapidly evolving through duplication and inversion events. A dominant mutation in the LRR domain of an *RPP39* homolog in Ws-0, *uni-1D* (named after the Japanese word for sea urchin because of its morphological phenotype) was found to display several defects in growth and hormone signaling which are often seen with gain of function R proteins such as SNC1 [47], [48]. Interestingly, the *uni-1D* phenotype was not dependent on NDR1 [47]. We found that RPP39 requires NDR1 to fully suppress *P. syringae* growth, but activates hypersensitive cell death independently of NDR1. In future, it will be interesting to more completely dissect the RPP39 signaling pathway.

Taken together, our results show the feasibility of employing computational predictions in the identification of functional pathogen effectors. In combination with classical genetic methods it was possible to determine function for one member of the large family of predicted R proteins in Arabidopsis. Preliminary data on several polymorphic effectors from our HMM priority list indicate that *Hpa* isolates other than Emoy2 contain functional/recognized effector alleles, which will be further pursued and may lead to the identification of additional *R* genes, the analysis of which will further advance our understanding of R protein signaling.

## Materials and Methods

### HMM prediction of effectors from *Hpa* Emoy2

A set of confirmed oomycete effectors and their close homologs used for the bioinformatic analyses included 43 sequences from *Phytophthora* species and *Hyaloperonospora arabidopsidis* and has been previously published [21]. The Signal Peptide and RxLR portion of these sequences (positions 1 to 90) were aligned using the *muscle* algorithm [49]. The HMM building, calibration, and searches were performed using the HMMER software package with *hmmbuild*, *hmmcalibrate*, and *hmmsearch* algorithms, respectively (http://hmmer.org/). The HMM search included only one iteration.

### Cloning of effectors

The predicted effectors were PCR amplified without signal peptide from *Hpa* strain Emoy2 DNA (obtained from Jonathan Jones) using primers listed in Table S2 and cloned into the pENTR/D-Topo vector (Invitrogen). The insert sequences were verified and the effectors recombined with LR clonase (Invitrogen) into the binary *Pseudomonas* expression vector pPsSP [35]. The pEDV3 clones of effectors were generated using restriction digests with SalI/BamHI or compatible restriction enzymes. Site-directed mutants ATR39-1^in^ and ATR39-2^del^ were generated in pENTR using the QuikChange Lightning kit (Agilent) and primers listed in Table S2, and recombined with binary vectors as above. For transient expression in *Nicotiana benthamiana*, *ATR39* alleles were recombined into pEarleygate201 containing a 35S promoter and N-terminal HA-tag [50]. The fasta files containing the amino acid and nucleotide sequences of the predicted effectors are available online as Text S1 and Text S2.

### Plant growth conditions

*A. thaliana* plants were grown on soil in controlled growth chambers at short days (8 hrs light/16 hrs dark cycle) and 24°C. Transgenic *Arabidopsis* were surface sterilized and selected on MS medium with the appropriate antibiotics. For *Hpa* growth assays the plants were transferred to a growth chamber with 18°C and 100% humidity.

### Pathogen assays

*Hpa* isolates were obtained from E. Holub (Maks9), X. Dong (Emwa1), J. McDowell (Emoy2, Emco5), X. Li (Cala2) and J. Jones (Noco2) and maintained on susceptible *Arabidopsis* plants as previously described [27]. For disease assays, conidiospores were harvested by vortexing in water, adjusted to 5*10^4^ spores/mL and sprayed on 2-week-old *Arabidopsis* seedlings. The infected plants were kept in growth chambers at 18°C and 100% humidity for 7 days before being stained with lactophenol-trypan blue [40] to assess *Hpa* growth or resistance.

*Pseudomonas fluorescens* strains were grown on Pseudomonas agar with glycerol (PAG) supplemented with the appropriate antibiotics. For HR assays, *Pf0* strains were grown on PAG plates for 2 days and resuspended in 10 mM MgCl_2_ to OD_600 nm_ = 1 (corresponding to 10^9^ cfu/mL). Bacteria were inoculated into halves of pierced *Arabidopsis* leaves using a blunt syringe. Visible HR symptoms were scored 24 hours post-inoculation. *Pseudomonas* growth assays were performed as described previously [51].

### Mapping and sequencing of the *RPP39* locus

Markers and probes used in map-based cloning of *RPP39* are summarized in Table S3. A fosmid library of Wei-0 was generated following the instructions in the copy control fosmid kit (Epicentre) and screened with Digoxigenin-labeled probes using the DIG DNA labeling and detection kit (Roche). To sequence the *RPP39* region, 350 bp-sized fragments of overlapping fosmids were sequenced in a 60 bp paired-end sequencing run on an Illumina G2. Reads were cleaned for vector sequences and bacterial contamination using MAQ (http://maq.sourceforge.net/) and assembled using CLC genomics workbench (http://www.clcbio.com/). Gaps and misassembled regions were filled in using Sanger sequencing data.

### Cloning and expression of *RPP39*

Genomic fragments of about 6 kb length, containing *RPP39 (R190*_Wei-0) or *R180*_Wei-0 were amplified using primers specified in Table S2 and introduced into pENTR/D-Topo (Invitrogen). Sequences were confirmed and the fragments recombined into pEarleygate301 [50] for expression in *Agrobacterium*. The *RPP39* cDNA clone was amplified from Wei-0 cDNA and recombined into pEarleygate100 containing a 35 S promoter [50]. The binary vectors were mobilized into *Agrobacterium tumefaciens* strain GV3101 with tri-parental mating and used to transform *Arabidopsis* plants following the floral dip protocol [52]. Presence of the transgene in *Arabidopsis* was confirmed by PCR. Transient expression experiments in *N. benthamiana* were performed as previously described [35]. Protein expression was determined by Western blotting as described [31].

### RNA extraction and RT–PCR

RNA was extracted from *Arabidopsis* seedlings infected with *Hpa* using the RNeasy plant mini kit (Qiagen) and reverse transcribed using Superscript III (Invitrogen) and oligo-dT primers. RT-PCR was performed with gene specific primers (Table S2) and 25 amplification cycles.

## Supporting Information

Figure S1 Growth assay with *Pseudomonas syringae* pv. *maculicola* ES4326. Bacterial titer was determined at 0 and 4 days post-infection. Significant differences as determined by Student's t-test are indicated by small letters (P<0.0001).
(TIF)

Figure S2 The effector domain of ATR39-1 is sufficient to trigger HR in Wei-0. A) Domain structure of ATR39. Numbers indicate amino acid residues. B) *Pf*0 inoculations of indicated ecotypes with ATR39-1 constructs. The lower halves of the leaves are inoculated with empty vector controls. Pictures were taken 24 hpi.
(TIF)

Figure S3 *ATR39-1* expression timecourse during *Hpa* Emoy2 infection. RT-PCR was performed on RNA extracted from infected tissue at the indicated timepoints. *Hpa* Actin was included as control for *Hpa* growth.
(TIF)

Figure S4 Dot matrix view of a pairwise alignment between the *RPP39* region in Wei-0 and Col-0 showing similarity between the two sequences. The genomic regions were aligned with Blast (blast2seq). Regions with gene duplications are visible at 20 k (corresponding to a gene family) and 70–80 k (corresponding to the *R* gene locus containing *RPP39*). Two gaps in the alignment (indicated by arrows) correspond to a transposable element in Wei-0 (at 64 k) and a translocation from Chromosome 3 (at 120 k).
(TIF)

Figure S5 Pairwise comparison of RPP39 with its homologs in Col-0 and Wei-0 and the more distantly related RPS5, RFL1 and RPS2. Depicted are % identities between the nucleotide (upper diagonal) and amino acid (lower diagonal) sequences of RPP39, its homologs R180_Wei-0, At1g61180_Col-0 and At1g61190_Col-0 as well as the R proteins RPS5, RFL1 and RPS2. The comparison is based on ClustalW alignments generated with CLC genomics workbench.
(TIF)

Figure S6 Amino acid alignment of RPP39 and its homologs from Wei-0 (R180-Wei-0) and Col-0 (At1g61180 and At1g61190). The alignment was performed using ClustalW and shaded in CLC genomics workbench.
(TIF)

Table S1 *Arabidopsis thaliana* ecotypes used in this study.
(DOC)

Table S2 Primers used in this study.
(DOC)

Table S3 Markers and probes used to map *RPP39*.
(DOC)

Text S1 Fasta formatted amino acid sequences of 18 top-scoring predicted effectors from *Hpa* strain Emoy2. The predicted signal peptide (by SignalP3.0) and RxLR motif are highlighted in blue and red, respectively.
(DOC)

Text S2 Fasta formatted nucleotide sequences of the top scoring effectors. Signal peptide sequences (not included in the cloned effectors) are highlighted in blue.
(DOC)
