# Supplementary figures and images for: Computational Prediction and Molecular Characterization of an Oomycete Effector and the Cognate Arabidopsis Resistance Gene
Source: PLoS Genet. 2012 Feb 16;8(2):e1002502. doi: 10.1371/journal.pgen.1002502 (PMC3280963; doi:10.1371/journal.pgen.1002502)

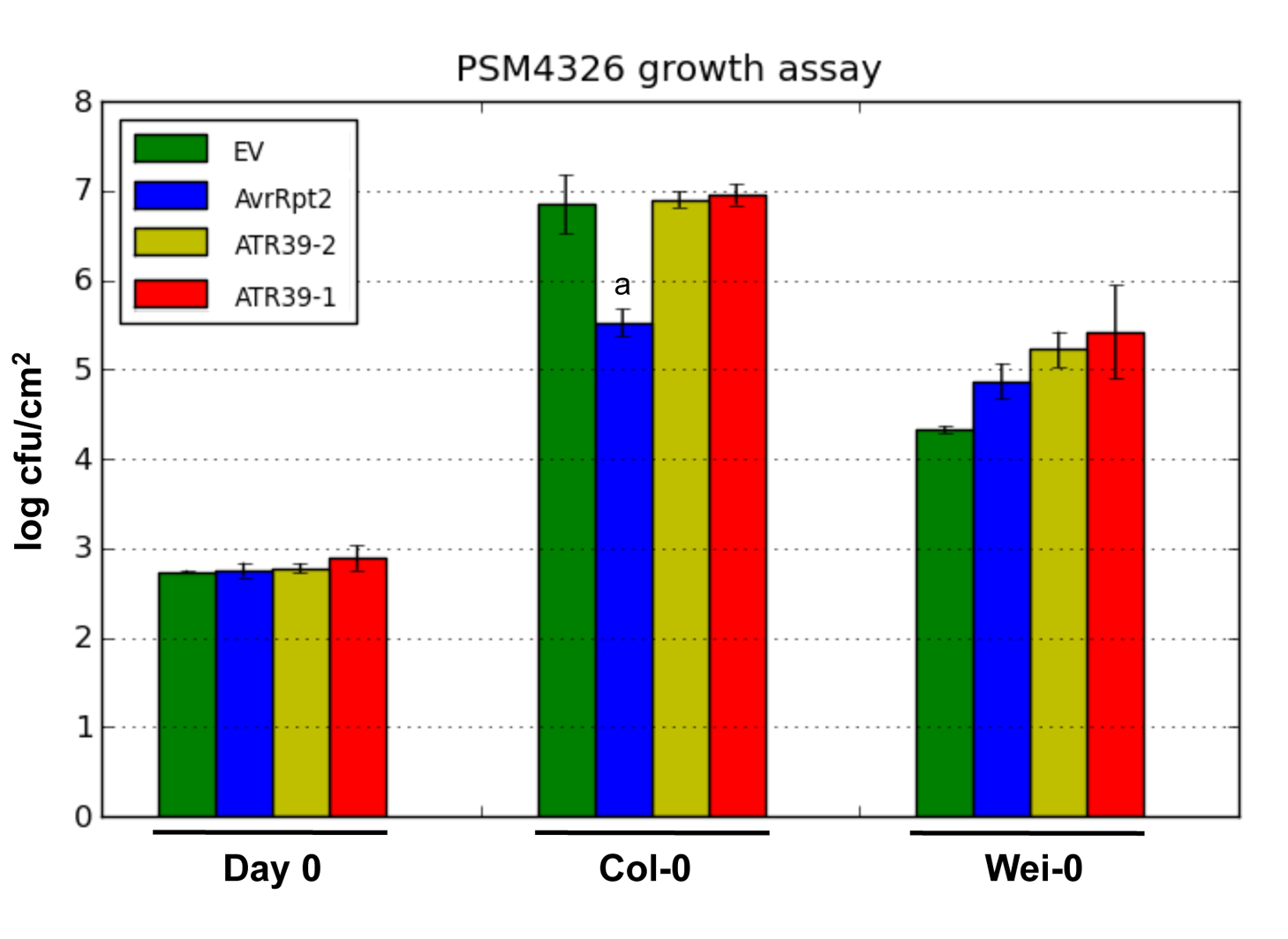

Supplement: Figure S1 — Growth assay with Pseudomonas syringae pv. maculicola ES4326. Bacterial titer was determined at 0 and 4 days post-infection. Significant differences as determined by Student's t-test are indicated by small letters (P<0.0001). (TIF) [file pgen.1002502.s001.tif]

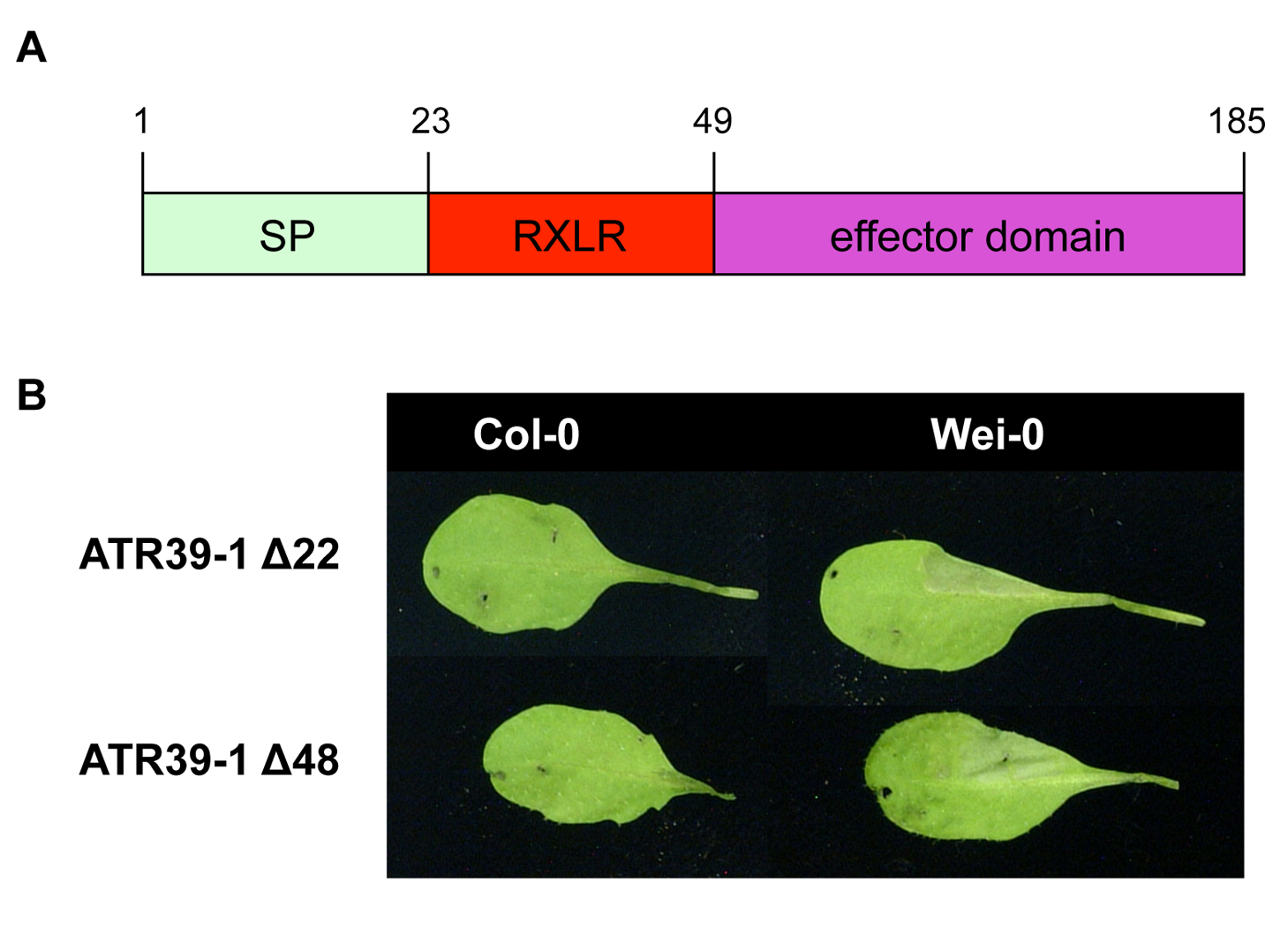

Supplement: Figure S2 — The effector domain of ATR39-1 is sufficient to trigger HR in Wei-0. A) Domain structure of ATR39. Numbers indicate amino acid residues. B) Pf0 inoculations of indicated ecotypes with ATR39-1 constructs. The lower halves of the leaves are inoculated with empty vector controls. Pictures were taken 24 hpi. (TIF) [file pgen.1002502.s002.tif]

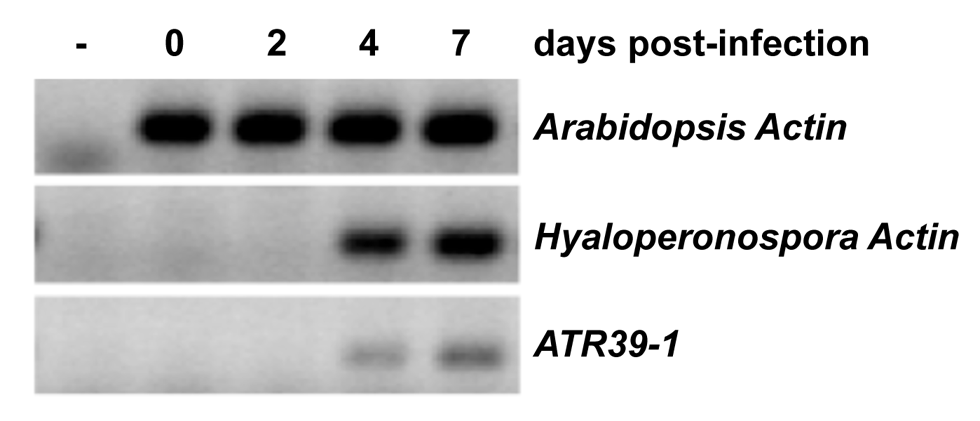

Supplement: Figure S3 — ATR39-1 expression timecourse during Hpa Emoy2 infection. RT-PCR was performed on RNA extracted from infected tissue at the indicated timepoints. Hpa Actin was included as control for Hpa growth. (TIF) [file pgen.1002502.s003.tif]

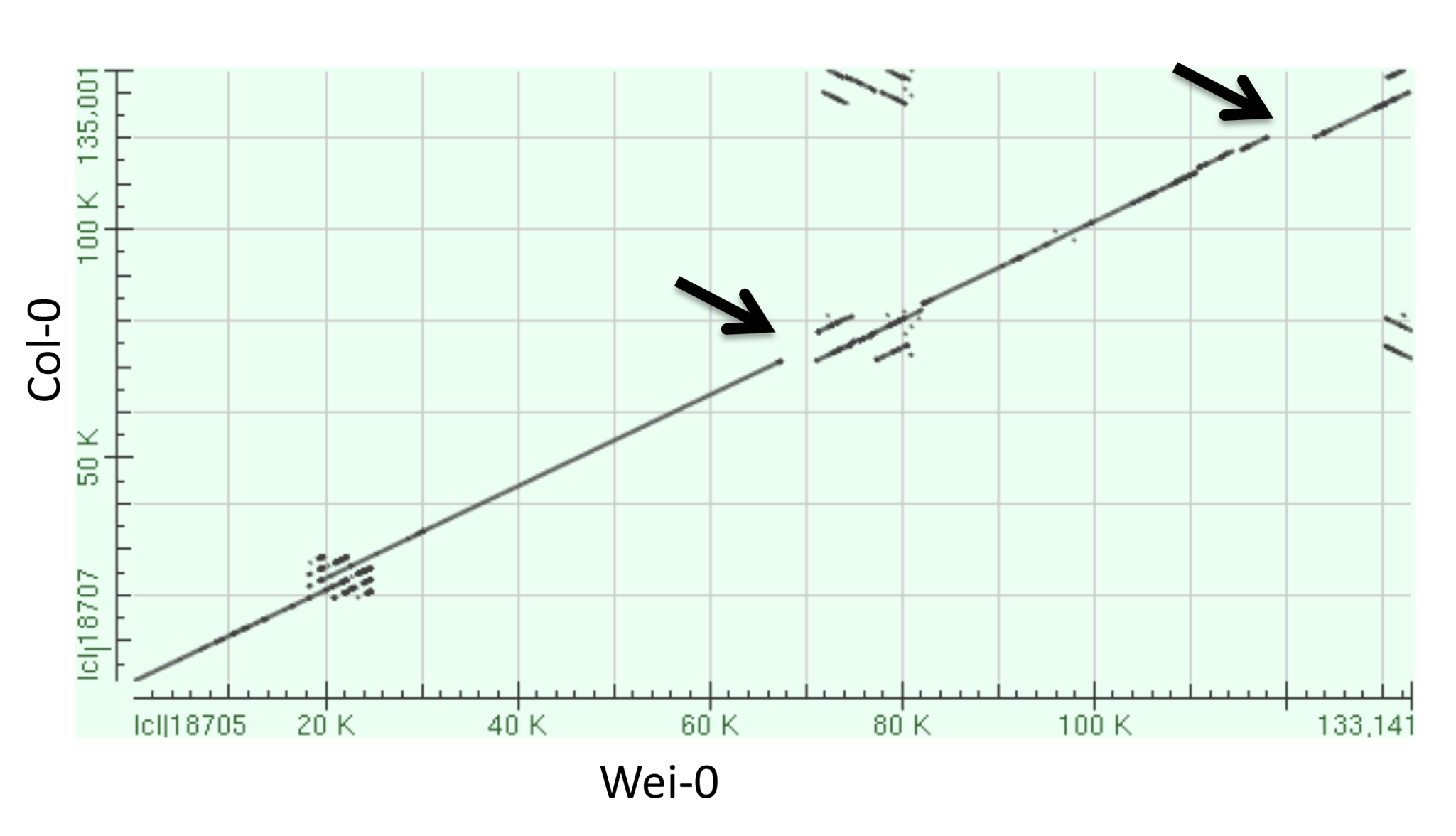

Supplement: Figure S4 — Dot matrix view of a pairwise alignment between the RPP39 region in Wei-0 and Col-0 showing similarity between the two sequences. The genomic regions were aligned with Blast (blast2seq). Regions with gene duplications are visible at 20 k (corresponding to a gene family) and 70–80 k (corresponding to the R gene locus containing RPP39). Two gaps in the alignment (indicated by arrows) correspond to a transposable element in Wei-0 (at 64 k) and a translocation from Chromosome 3 (at 120 k). (TIF) [file pgen.1002502.s004.tif]

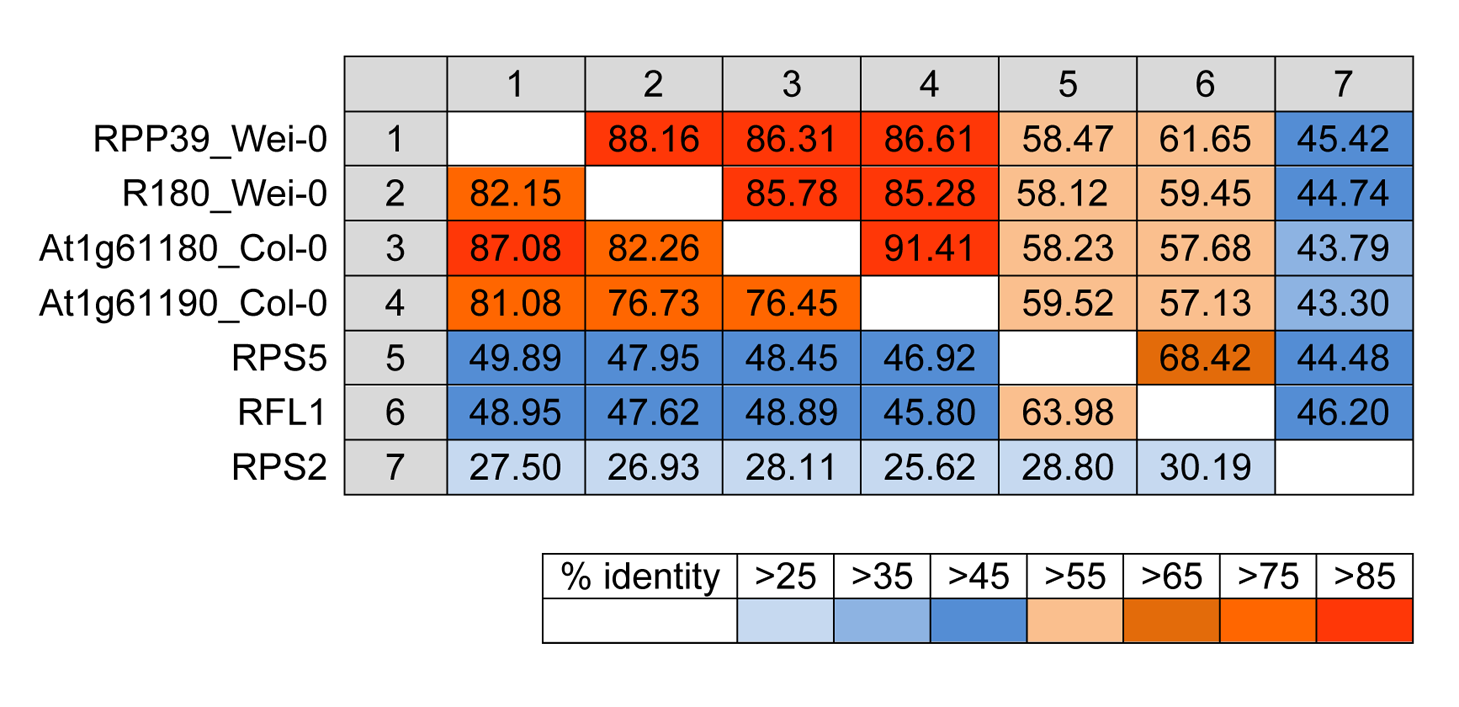

Supplement: Figure S5 — Pairwise comparison of RPP39 with its homologs in Col-0 and Wei-0 and the more distantly related RPS5, RFL1 and RPS2. Depicted are % identities between the nucleotide (upper diagonal) and amino acid (lower diagonal) sequences of RPP39, its homologs R180_Wei-0, At1g61180_Col-0 and At1g61190_Col-0 as well as the R proteins RPS5, RFL1 and RPS2. The comparison is based on ClustalW alignments generated with CLC genomics workbench. (TIF) [file pgen.1002502.s005.tif]

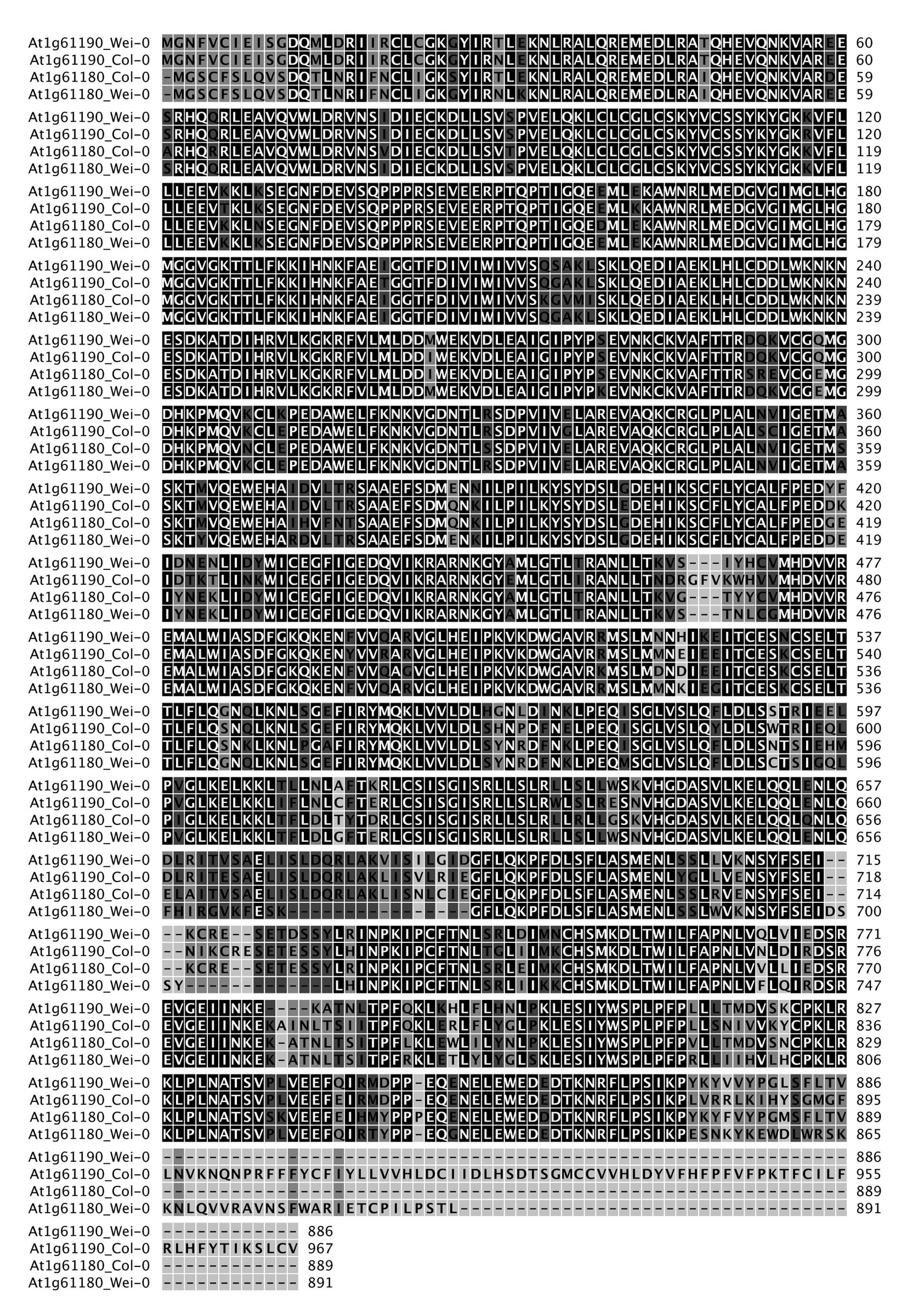

Supplement: Figure S6 — Amino acid alignment of RPP39 and its homologs from Wei-0 (R180-Wei-0) and Col-0 (At1g61180 and At1g61190). The alignment was performed using ClustalW and shaded in CLC genomics workbench. (TIF) [file pgen.1002502.s006.tif]
