# Supplementary material for: Computational Prediction and Molecular Characterization of an Oomycete Effector and the Cognate Arabidopsis Resistance Gene
Source: PLoS Genet. 2012 Feb 16;8(2):e1002502. doi: 10.1371/journal.pgen.1002502 (PMC3280963; doi:10.1371/journal.pgen.1002502)
Supplement: Table S1 — Arabidopsis thaliana ecotypes used in this study. (DOC) [file pgen.1002502.s007.doc]

Supplemental Table 1: *Arabidopsis thaliana* ecotypes tested in this study

| ID | Origin | ABRC stock number | ID | Origin | ABRC stock number |
| --- | --- | --- | --- | --- | --- |
| Ag-0 | France | CS22-630 | Nd-1 | Germany | CS22-619 |
| An-1 | Belgium | CS22-626 | NFA-10 | England | CS22-599 |
| Bay-0 | Germany | CS22-633 | NFA-8 | England | CS22-598 |
| Bor-1 | Czech | CS22-590 | Nok-3 | Netherlands | CS22-643 |
| Bor-4 | Czech | CS22-591 | Omo2-1 | S Sweden | CS22-584 |
| Br-0 | Czech | CS22-628 | Omo2-3 | S Sweden | CS22-585 |
| Bur-0 | Ireland | CS22-656 | Oy-0 | Norway | CS22-658 |
| C24 | Portugal | CS22-620 | Pna-10 | USA | CS22-571 |
| CIBC-5 | England | CS22-602 | Pna-17 | USA | CS22-570 |
| Col-0 | USA | CS22-625 | Pro-0 | Spain | CS22-649 |
| CS22491 | Russia | CS22-621 | Pu2-23 | Croatia | CS22-593 |
| Ct-1 | Italy | CS22-639 | Pu2-7 | Croatia | CS22-592 |
| Cvi-0 | Cape Verde | CS22-614 | Ra-0 | France | CS22-632 |
| Eden-1 | N Sweden | CS22-572 | Ren-1 | France | CS22-610 |
| Edi-0 | Scotland | CS22-657 | Ren-11 | France | CS22-611 |
| Ei-2 | Germany | CS22-616 | Rmx-A02 | USA | CS22-568 |
| Est-1 | Estonia | CS22-629 | Rmx-A180 | USA | CS22-569 |
| Fei-0 | Portugal | CS22-645 | RRS-10 | USA | CS22-565 |
| Ga-0 | Germany | CS22-634 | RRS-7 | USA | CS22-564 |
| Got-22 | Germany | CS22-609 | Se-0 | Spain | CS22-646 |
| Got-7 | Germany | CS22-608 | Shakdara | Tajikistan | CS22-652 |
| Gu-0 | Germany | CS22-617 | Sorbo | Tajikistan | CS22-653 |
| Gy-0 | France | CS22-631 | Spr1-2 | S Sweden | CS22-582 |
| HR-10 | England | CS22-597 | Spr1-6 | S Sweden | CS22-583 |
| HR-5 | England | CS22-596 | Sq-1 | England | CS22-600 |
| Kas-2 | Kashmir | CS22-638 | Sq-8 | England | CS22-601 |
| Kin-0 | USA | CS22-654 | Tamm-2 | Finland | CS22-604 |
| Knox-10 | USA | CS22-566 | Ts-1 | Spain | CS22-647 |
| Knox-18 | USA | CS22-567 | Ts-5 | Spain | CS22-648 |
| Kondara | Tajikistan | CS22-651 | Tsu-1 | Japan | CS22-641 |
| Kz-1 | Kazakhstan | CS22-606 | Ull2-3 | S Sweden | CS22-587 |
| Kz-9 | Kazakhstan | CS22-607 | Ull2-5 | S Sweden | CS22-586 |
| Ler-1 | Poland | CS22-618 | Uod-7 | Czech | CS22-613 |
| LL-0 | Spain | CS22-650 | Van-0 | Canada | CS22-627 |
| Lov-1 | N Sweden | CS22-574 | Var2-6 | S Sweden | CS22-581 |
| Lov-5 | N Sweden | CS22-575 | Wa-1 | Poland | CS22-644 |
| Lp2-2 | Czech | CS22-594 | Wei-0 | Switzerland | CS22-622 |
| Lz-0 | France | CS22-615 | Ws-0 | Ukraine | CS22-623 |
| Mr-0 | Italy | CS22-640 | Ws-2 | Ukraine | CS22-659 |
| Mrk-0 | Germany | CS22-635 | Wt-5 | Germany | CS22-637 |
| Ms-0 | Russia | CS22-655 | Zdr-1 | Czech | CS22-588 |
| Mt-0 | Libya | CS22-642 | Zdr-6 | Czech | CS22-589 |
| Mz-0 | Germany | CS22-636 |  |  |  |
