# Supplementary material for: Computational Prediction and Molecular Characterization of an Oomycete Effector and the Cognate Arabidopsis Resistance Gene
Source: PLoS Genet. 2012 Feb 16;8(2):e1002502. doi: 10.1371/journal.pgen.1002502 (PMC3280963; doi:10.1371/journal.pgen.1002502)
Supplement: Table S2 — Primers used in this study. (DOC) [file pgen.1002502.s008.doc]

Supplemental Table 2: Primers used in this study.

| **Primername** | **Sequence** | **purpose** |
| --- | --- | --- |
| Hp463.5-SP F | caccGTCCCGACTGCTGGTAATCC | amplify effectors from Emoy2 |
| Hp463.5_Spe1 R | TCAactagtTCTCTGGCTTGTATCATG | amplify effectors from Emoy2 |
| Hp96.3 -SP F | caccGTCCCGACTGCTGG | amplify effectors from Emoy2 |
| Hp96.3_Spe1 R | CTAactagtGCCATCCTTGTGACCCTG | amplify effectors from Emoy2 |
| Hp192.1-SP F | caccGTCTCGACTGCTGTCG | amplify effectors from Emoy2 |
| Hp192.1_Spe1 R | CTAactagtTTGTAGACAAGCGG | amplify effectors from Emoy2 |
| Hp450.9-SP F | caccAGCTACGTTGCGGATGCC | amplify effectors from Emoy2 |
| Hp450.9_Spe1 R | TTAactagtAGCTGGATAAAATGAC | amplify effectors from Emoy2 |
| Hp258.6-SP F | caccGGCTTAGTTCCTTCAAGG | amplify effectors from Emoy2 |
| Hp258.6_Spe1 R | TCAactagtTCCATGCTTTTGCG | amplify effectors from Emoy2 |
| Hp388.1-SP F | caccGCCACGGAGGCTGCTGG | amplify effectors from Emoy2 |
| Hp388.1_Spe1 R | TCAactagtGCTTTGATAACAGGC | amplify effectors from Emoy2 |
| Hp4150.1-SP F | caCCGTGTCCACGGCCAC | amplify effectors from Emoy2 |
| Hp4150.1_Spe1 R | TTAactagtCCTACCATGACCCG | amplify effectors from Emoy2 |
| Hp750.2-SP F | caccTTGGTGCGGACGGAGAC | amplify effectors from Emoy2 |
| Hp750.2_Spe1 R | TTAactagtGTTACGATATTTAATATCCG | amplify effectors from Emoy2 |
| Hp904.6-SP F | caccCTGCCAGCTCGCGTAGCC | amplify effectors from Emoy2 |
| Hp904.6_Spe1 R | TTAactagtAATCGCCGGATTGATGCCG | amplify effectors from Emoy2 |
| Hp214-SP_Sal1 F | CACCGTCGACATGCGTGACACAAACTC | amplify effectors from Emoy2 |
| Hp214.3-Bgl2 R | TGCagatctGTTGAGCGTCAACATAAG | amplify effectors from Emoy2 |
| Hp204.2-SP F | caccATGATTATGATCACCAATGC | amplify effectors from Emoy2 |
| Hp204.2_Spe1 R | TTAactagtTTCTCGGACTGTG | amplify effectors from Emoy2 |
| Hp550.2-BamH1 R | TGCGGATCCCGATGCCGGATTCG | amplify effectors from Emoy2 |
| Hp550.2-SP_Sal1 F | caccGTCGACATGGAGGTGGTCCTGATC | amplify effectors from Emoy2 |
| Hp2080.1-SP_PspX1 F | caccATGCTCGAGGCCCCGGAAGTCAC | amplify effectors from Emoy2 |
| Hp2080.1_BamH1 R | TGCGGATCCAGTTCCTGGGGGCTTCAG | amplify effectors from Emoy2 |
| Hp166.8_BamH1 R | TGCGGATCCCGGTACTAGATGAATGCC | amplify effectors from Emoy2 |
| Hp166.8-SP_Sal1 F | caccGTCGACATGCGTACGCTTAATGATG | amplify effectors from Emoy2 |
| ATR39-SP_F | caccATGGACCCAACAGTCAAAAGAC | amplify effectors from Emoy2 |
| ATR39_Spe1-R | TTAactagtTTTGTTACCCAGATGTATG | amplify effectors from Emoy2 |
| ATR39-RXLR_F | CACCatgGCGTCCGATGTCC | amplify ATR39 without RXLR |
| ATR39.1-spec_R | ACCACATCTATATCCGAC | ATR39-1 specific reverse primer |
| ATR39.2-spec_R | TCACCACATCTACATCTACC | ATR39-2 specific reverse primer |
| Hpa Act2 Fw | GCCCCAAACACTTA | RT-PCR control |
| Hpa Act2 Rev | GCCGGCACATTGAACG | RT-PCR control |
| ATR39.2_delEV_R | CCTCTATTCACCACATCTATATCCGGCAATGTTAATTTC | delete E168V169 from ATR39-2 |
| ATR39.2_delEV_F | GAAATTAACATTGCCGGATATAGATGTGGTGAATAGAGG | delete E168V169 from ATR39-2 |
| ATR39.1_inEV_F | GAGATTTACATTGTCGGATGTAGATATAGATGTGGTGAATAGAGG | insert E168/V169 in ATR39-1 |
| ATR39.1_inEV_R | CCTCTATTCACCACATCTATATCTACATCCGACAATGTAAATCTC | insert E168/V169 in ATR39-1 |
| R180_Wei-0_F | caccTAACGGTGATAATGACGG | amplify genomic fragment of R gene candidate |
| R180_Wei-0_R | CCATGGTTGAAGGAAAGAGAG | amplify genomic fragment of R gene candidate |
| R190_Wei-0_F | cacCATCTAGTCTCAATCTCTGG | amplify genomic fragment of RPP39 |
| R190_Wei-0_R | AGAACCAATAACCGGATCATG | amplify genomic fragment of RPP39 |
| RPP39_cDNA_F | caccATGGGAAATTTTGTGTGT | amplify RPP39 cDNA |
| RPP39_cDNA_R | TGCTCTAGAGACAGTGAGGAAACTCAG | amplify RPP39 cDNA |
