# Supplementary material for: Computational Prediction and Molecular Characterization of an Oomycete Effector and the Cognate Arabidopsis Resistance Gene
Source: PLoS Genet. 2012 Feb 16;8(2):e1002502. doi: 10.1371/journal.pgen.1002502 (PMC3280963; doi:10.1371/journal.pgen.1002502)
Supplement: Table S3 — Markers and probes used to map RPP39. (DOC) [file pgen.1002502.s009.doc]

Supplemental Table 3: Markers and probes used in mapping of RPP39.

| **Name** | **Position** | **polymorphism Col-0/Wei-0** | **Used as** | **Forward primer** | **Reverse primer** |
| --- | --- | --- | --- | --- | --- |
| T14L22 | 19.23 MB | indel | marker | ttatattcccaatggcttgg | aagcaaactcacctcctag |
| F19K23 | 22.95 MB | indel | marker | gtcatcatgttgttcacaag | atgcacgtaaagacttggg |
| T13D8 | 22.25 MB | indel | marker | tgtcgttagaactagcttc | cacaagtcagtgatcaaac |
| T7P1 | 22.49 MB | CAPS -/AluI | marker | acgaggttatacgcaatacc | atgttgttcccgatcatgtc |
| T13M11 | 22.79 MB | CAPS MslI/- | marker | tgtctggtaaaccttcgag | acatatgtgtcagatgcaac |
| M22.58 | 22.58 MB | several SNPs | marker/probe | atcatgtagcgtgtatcacg | aagccttctttctcgagtag |
| At1g61100 | 22.51 MB | several SNPs | probe | aatatgtctccttcacgtc | aatagaattctcaatctgc |
| RPP-probe | 22.55/22.65 MB | probe for fosmid library | probe | caccatggggagttgttttct | acgctagcatctccatg |
