# Supplementary material for: Computational Prediction and Molecular Characterization of an Oomycete Effector and the Cognate Arabidopsis Resistance Gene
Source: PLoS Genet. 2012 Feb 16;8(2):e1002502. doi: 10.1371/journal.pgen.1002502 (PMC3280963; doi:10.1371/journal.pgen.1002502)
Supplement: Text S1 — Fasta formatted amino acid sequences of 18 top-scoring predicted effectors from Hpa strain Emoy2. The predicted signal peptide (by SignalP3.0) and RxLR motif are highlighted in blue and red, respectively. (DOC) [file pgen.1002502.s010.doc]

>Hp_Contig137.3_F55

MRVCYFVLVPSVALAVIATESSETSGTIVHVFPLRDVADHRNDALINRALRAQTALDDDEERWPFGPSAVEALIETIDRHGRVSLNDEAKMKKVVRTWKKLIERDDLIGEIGKHYFEAPGPLHDTYDEALATRLVTTYSDRGVARAILHTRPSDPLSKKAGQAHRLEEAVASLWKGRGYTSDNVVSSIATGHDVDFFAPTAFTFLVKCVESEDDANNAIFEYFGSNPSRYFSAVLHAMEKPDADSRVLESSKKWMFQCYAQKQFPTPVFERTLAAYQSEDYAIRGARNHYEKLSLSQIEELVEEYSRIYSVZ

>Hp_Contig1514.4_F2

MRLVHAVLLPGIIVFVSNGNLLHAHALHEDETGVTAGRQLRAAASEVFGLSRASFGLGKAQDPLDKFFRKIINSRKPIETSYSAKGIHEKIIKAYDRHVFESKKAHDRHVSKSKKAHGRHVSKSKMAHDRHVSKSEKAPIQYASVADYLKKIYPGTDIERIVSTLKRHDEVGAKDLGAKLGTAVASQZ

>Hp_Contig166.8_F9

MRLLHPVLLPIVIVFASNTKLLHARTLNDDETDVSVRRFLRSNTAEAGRVDGQSTSSVARFIDRVFGKSASAPKATTVTTEAQVTAGEAAQKARENAEKSLKDRLRPLLSEKGGALVNSKGSSVFAEMVKAVDHVNKKLPHDHAISLDKFLIGTVEHKRLVELATTGAESTKKEVSNKASKLLEKVAAENGIHLVPZ

>Hp_Contig192.1_F23

MRLPYLALTALVAVFSSGDAVSTAVGSESVYESDVPLVLCETDESVNRANTQRFLRATVVTRDSVSMPTIMEEGMVTFPSVLCMSFSATIEKVKFSMATLEKGGKATEAMITVMSNLAESIRQATGSQAEELGLGVIIYKALHEHRNQHVFVDRVSRNKTRKELLKRIRIDHVTACLQZ

>Hp_Contig204.2_F52

MRVYCFVLVPSVAVAIMITNALETPGSSDLLAIARDATDRRNAIPTNRSLRAHEGTDHEERIITRQFGNWLRKLFPIPSTSLGGSSKVKELFYSTAQQAKGKDKIVTDVKDLRGAMQFLETLDNRVQLKAFQKLCVRRKSGDEVAKSRDEVAAGLAGKYGDAAMAFALYRRAPSTMGKKSAAFKSVRDVMTARWKANAEITPEMIYKWLTPNFPLTDEDLFKPAFTELLYGISSDEETAHKFLWTYLRGDYGRIGQVQFTSAGYDHIDKQAVRKFTNWVNVEHPRFTVREZ

>Hp_Contig2080.1_R17

MRPPIILLASTAVSVCITGAPEVTNSSTKEADISSSVPFVGDDSINAPANRRLRAHDAVGGEERAPVPSSLTTEILKKTELAVRKLYDGTNRPNSLSMHAWRSVINADSKIMKHVIAVAERKNQRVPQQTPNGETTGDDMSGLVLTAQANHLQGEIDKLPIMKWMTGKTTGELVDVLKEHKFYNTFSYTRVQALTVVLENFNDAMKTKIKLFDTLVGVYGGVAKYARVLSIAKAGVLTGKMILPLQTELVKSLHWETLDDLLVLLKLDLKRDSLTYEALDTVVTCAALRYKLSVDDALYATFTGLRAMYTDEIVKDAIKKGRGEIISA

>Hp_Contig214.3_F41

MRLISFALATSTAILARDTNSSRTRGSTVTNASLPAIFRSSVGNHNDVVVKRLLRAREIAADEERTPRKLPSFDKVISELFATLHVEETYPPDLGKKIINKLRTFDREAIKHYYEKQYEDPIMATKKLIEASSLKHQRTHGPFDMEMYREFYHDHLHKETWISHWVQDGLHKAELHPNVVFKMMINAEKRPVLGADSRSLFATTDLEALHKYIERFNEKEKSRTPASLRQTLSYCIRDEAGLASFLSIAKQNSINAPFVWKEQHRLFMGWIGHRKTIDQVADMMKIPGQWKDAKACPFLDTLIGYVTVFAQTYPAASTDIVSCLVIKFGHLYAAMLIGEAKEVNIDVFAELEKVLFQSWTKGGTNPLNFDQADFFAEITVGADDKALIREHFAEHYRKETPSHLMLTLNZ

>Hp_Contig258.6_R51

MRFTVAAFAAAAAIACSTSDVTPVPGAPYRSPTLLTRDALHDGKGARFLRGYETDAAAINNNNAEERSVTALSALHPNPTLKNKELPAAFKAPIRFVVGLIRRIGAEVRLMTQRRKRIQPHDPLLAGIGZ

>Hp_Contig388.1_F27

MRFIALTLVASTALLPRTESATEAAGSSTTDQRDAAVVVHVTSDYYGSLSSRLLRSQVGPSEVLESAISAVASRIKPAKSFFGIGTARVRRSIFHVAGRWYSHGDQGACYQSZ

>Hp_Contig399.11_F1

MVKCTPLLALTVIVSAGSDALSDPTVKRLAKLATINQAPATQSNSDSKRVLRASDVPDEVAAGESRSPKSLWPWEVEDKLAPLKEKLISTSADDLEAGGSANKNALPMIWRWLWQNQIETKKTPIDEISKEVQTAMDLISSQATHEELNKAGVSVSDYVKALRFTLSDIDVVNRGMEYNIHLGNKZ

>Hp_Contig399.12_F6

MVKCTPLLALTVIVSAGSDALSDPTVKRLAKLATINQAPATQSNSDSKRVLRASDVPNEVAAGESRSPKSLWPWEVEDKLAPLKEKLISTSADDLEAGGSAKKNALPMTWRWLWQNQIETKKTPIDEISKEVQTAMDLISSKATHEELNKAGVSVSDYVKALKLTLPEVDVDVVNRGMEYNIHLGNKZ

>Hp_Contig4150.1_F1

MRITSVYVAIVAASLHAIGSAVSTATNVGATVLENKIPAAIAPSLRTSEGRSLRTEEKRGQSDLGEERGRGNLPTTDSFSASLKRFIRALFCMGSKKGMTRTKSSGSSIVSGHGRZ

>Hp_Contig450.9_F29

MRLICVLLLAVVSQMTVSSYVADAKDVTKPANQGTGSEQLKARRYLRDDKGTFGDQNTAMNNKDEERIFEGVFNKLMALFHGSIDAKQVKPLIQETHILEENVANLQAIPTASKKGLSQFFAENPAIKKEIIVAGVLLSLIVAVPLTVKSFYPAZ

>Hp_Contig463.5_F20

MRLTYTALATLAALLSCSDSVPTAGNPKAVLKHDVSLVARGTGGDDNGADGKRLLRNAHAQDESQDSQLFEERSVHVPEKATVVVASGVAHVIPSEASTIAKKVVEANTHVKDGAGQEKYWLLTKLESFKKKLMESRKFQAIMEKLNTFLGRKPTTTAGNTNTVKDEGKATANGGTHTTHDDKPPVTTIIHPEAGGQSKFASTRGAVAERMKDATKKTQDKVAEWNKKLEQYKKKVKEDPKFQAIMEKLNTFLGRKPTTTAGNTNTVIDEGKATANGGTHTTHDTSQRZ

>Hp_Contig550.2_F9

MRLSAILLLIVAPLHLCVGEVVLIPATMENPLLRSAPSTNADARKGARSLRALNSAGISQLLEPFTTKVKSFVPRTAAYAAAKQARVKKAAAARTAQIEQKSEENQAALLKMGLAAFGQDVSSAIVKANSQNSFFQRDGSFFLILFKRGKSVDDLTGMLESACQTSDISVEVAVRDTVRQYKLYREDPKRREISFLLANPASZ

>Hp_Contig750.2_F4

MRIHVRVLLGVAALVVGIHTTVALVRTETTNEDTQLSGRIHSTGDNDVSCRRFLRTNDAHAKDEEERVDTKAVSAWVQKVLSKLKTSALIDWHLILDHDPGYVRGKYPNDKVLGDKYYARWADIKYRNZ

>Hp_Contig904.6_F2

MTKCSLLLVPFLVAIAVSDALPARVAGTLPQSATSVQDKATESTVSGKRALRSKKDTKGAADEERALLSPSILEPLSTKMKSSTDWMAQTRKGASFSVSGVSQKELGESKDVVVRLENLQRDFQKSTEQMTIDMARDLTFSGATRKVGRGITWLPLNRVLQDRKYREWLETMSKRDAYAAKLEELIAAAKKRVDGINPAIZ

>Hp_Contig96.3_R9

MRLTYTALATLAALLSCSDSVPTAGNPKAVLKHDVSLVARGTGGDDNGADGKRLLRNAHAQDESQDSQLFEERSVAVPEAATGAASAVVHSFTSEASPIAKNVVEATTHVKDGAGQEKNWLLTKLESFKDWAKELSFIKGIMKWWKRWIHNPNHVDKNAKPVSSTPSVEGEKITEKEAEVKKSATADKSLPPPPAYTPRLSEGNVKTAGETSKVPTDNGKLPVGTVTKTEGKSTVSSQNEKPIMTAGTHGTDSTKPQGAGNPSKNTEGGVPLKIDAPVSTQGHKDGZ
