# Supplementary material for: Computational Prediction and Molecular Characterization of an Oomycete Effector and the Cognate Arabidopsis Resistance Gene
Source: PLoS Genet. 2012 Feb 16;8(2):e1002502. doi: 10.1371/journal.pgen.1002502 (PMC3280963; doi:10.1371/journal.pgen.1002502)
Supplement: Text S2 — Fasta formatted nucleotide sequences of the top scoring effectors. Signal peptide sequences (not included in the cloned effectors) are highlighted in blue. (DOC) [file pgen.1002502.s011.doc]

>Hp_Contig137.3_ATR1

ATGCGCGTCTGCTACTTCGTTCTCGTGCCCTCTGTCGCTCTGGCCGTCAT

AGCCACCGAGTCGTCGGAAACGTCCGGCACGATCGTCCACGTGTTCCCCC

TCCGTGACGTCGCCGACCATCGCAACGACGCTCTAATCAATCGGGCGTTG

AGGGCGCAGACAGCTCTCGATGACGATGAAGAGAGATGGCCGTTCGGGCC

TTCGGCCGTCGAAGCGTTGATTGAAACCATCGACAGACATGGGCGAGTCA

GCTTGAATGATGAGGCTAAGATGAAGAAGGTCGTACGAACCTGGAAAAAA

CTAATCGAACGGGATGATTTGATTGGCGAGATAGGAAAACATTATTTTGA

AGCGCCGGGGCCACTCCATGATACCTATGATGAAGCTCTCGCCACTAGGC

TAGTAACAACCTATTCGGATCGCGGAGTAGCTCGGGCAATTCTACATACA

AGGCCTTCGGACCCACTTTCGAAAAAGGCAGGGCAAGCGCACCGATTGGA

GGAAGCAGTGGCATCACTATGGAAAGGGCGAGGATACACCTCAGACAATG

TCGTTTCAAGCATTGCTACCGGCCACGATGTTGACTTTTTCGCACCCACG

GCCTTCACTTTCCTAGTAAAGTGTGTCGAATCTGAGGACGACGCGAACAA

CGCCATATTCGAGTATTTTGGTAGTAATCCCAGCAGATACTTTTCCGCTG

TGCTGCACGCGATGGAGAAGCCTGATGCTGATAGTCGAGTGCTTGAGAGC

AGCAAAAAATGGATGTTTCAATGTTATGCGCAGAAGCAGTTCCCTACACC

AGTATTTGAAAGGACCCTGGCTGCTTACCAGTCTGAAGATTATGCGATTC

GTGGTGCACGGAACCACTACGAGAAGCTCAGCCTCTCTCAAATCGAGGAA

CTCGTGGAAGAGTATTCGAGAATATATTCTGTTTAA

>Hp_contig1514.4_ATR13

ATGCGCCTTGTTCACGCGGTACTGCTACCTGGCATTATTGTCTTCGTATC

CAACGGGAATCTGCTCCACGCCCATGCGCTCCATGAGGACGAGACGGGTG

TCACTGCTGGTCGCCAGCTCCGAGCAGCCGCCAGCGAAGTATTTGGACTC

TCACGAGCTAGTTTCGGGCTTGGTAAGGCTCAAGATCCGCTCGACAAGTT

CTTTAGGAAGATAATAAACTCGCGGAAGCCCATCGAAACCAGTTATTCGG

CTAAAGGCATCCACGAGAAGATTATAAAGGCATACGATCGTCATGTCTTC

GAATCTAAGAAGGCACACGATCGTCATGTCTCCAAATCTAAGAAGGCACA

CGGTCGTCATGTCTCCAAATCTAAGATGGCACACGATCGTCATGTCTCCA

AATCTGAGAAGGCCCCCATCCAGTATGCCAGTGTAGCGGATTACCTGAAA

AAAATCTACCCCGGTACGGACATTGAACGTATTGTCTCGACGCTCAAGCG

TCACGACGAAGTAGGAGCTAAAGATTTGGGAGCGAAACTTCAGACTGCCG

TTGCCAGTCAGTAG

>Hp_Contig166.8

ATGCGTCTACTCCATCCGGTGTTGCTACCTATCGTCATCGTCTTCGCGTC

CAACACGAAACTACTTCATGCTCGTACGCTTAATGATGATGAGACGGATG

TTTCGGTTCGTAGATTCCTCCGATCCAACACAGCAGAGGCCGGTCGGGTC

GATGGACAATCGACCAGTAGTGTCGCTCGTTTTATCGATCGCGTCTTCGG

GAAATCGGCTTCGGCGCCGAAAGCTACCACGGTAACTACGGAGGCTCAAG

TCACAGCTGGTGAGGCCGCTCAAAAAGCACGAGAGAATGCAGAGAAGAGT

TTAAAGGATCGACTCAGGCCTTTGCTTTCCGAAAAGGGTGGGGCTCTAGT

CAATTCCAAAGGTAGCTCAGTTTTTGCCGAGATGGTGAAAGCGGTTGACC

ACGTCAACAAAAAACTCCCCCATGACCATGCCATCTCTTTGGACAAGTTC

CTTATAGGCACAGTCGAACATAAGAGACTGGTTGAATTGGCTACCACAGG

GGCTGAGAGTACGAAAAAAGAGGTGAGCAACAAAGCCTCAAAGTTGCTCG

AAAAAGTTGCTGCTGAGAATGGCATTCATCTAGTACCGTAG

>Hp_Contig192.1

ATGCGTCTACCGTACCTTGCATTAACAGCTCTGGTCGCTGTTTTCTCTTC

AGGCGATGCGGTCTCGACTGCTGTCGGCTCGGAGTCTGTTTACGAGTCTG

ACGTCCCTCTCGTGCTTTGCGAGACGGATGAATCCGTCAACAGAGCAAAC

ACGCAGAGGTTTTTGAGAGCCACTGTTGTGACAAGAGATTCTGTCTCCAT

GCCTACTATTATGGAAGAGGGGATGGTGACTTTTCCAAGCGTACTTTGCA

TGTCCTTCTCTGCAACCATCGAAAAAGTCAAATTCAGTATGGCGACCTTG

GAGAAAGGCGGCAAAGCGACAGAAGCGATGATCACAGTGATGAGCAACCT

AGCGGAATCAATTCGGCAAGCGACGGGGTCCCAGGCGGAAGAGTTGGGTC

TTGGCGTGATCATCTACAAGGCCTTGCATGAACACCGTAATCAACATGTG

TTCGTGGACCGGGTTAGCCGTAATAAAACTCGCAAGGAGCTGTTGAAGCG

AATCCGAATCGACCATGTCACCGCTTGTCTACAA

>Hp_Contig204.2

ATGCGCGTGTACTGCTTCGTGCTCGTGCCTTCTGTCGCCGTTGCGATTAT

GATCACCAATGCGTTGGAAACGCCCGGTTCGAGCGACCTCTTGGCCATCG

CTCGTGACGCCACCGATCGTCGAAACGCCATCCCAACCAATCGGTCGTTG

AGGGCGCACGAAGGTACCGACCATGAAGAGAGAATCATCACCAGGCAGTT

TGGTAATTGGCTTCGCAAACTCTTTCCTATTCCTTCGACGTCGTTGGGTG

GTTCATCGAAAGTGAAAGAGTTGTTTTACAGCACCGCGCAACAGGCAAAG

GGCAAGGACAAGATCGTGACCGACGTTAAAGACTTGAGGGGGGCCATGCA

GTTCCTGGAGACGCTCGACAACCGAGTGCAATTAAAAGCCTTTCAGAAGC

TCTGCGTGAGACGCAAGTCTGGAGATGAGGTGGCCAAGTCTAGAGATGAG

GTGGCCGCTGGTCTAGCAGGCAAATATGGGGATGCCGCGATGGCTTTTGC

GCTTTACAGAAGGGCCCCGAGCACGATGGGAAAAAAAAGCGCCGCATTTA

AGTCTGTGCGAGACGTGATGACCGCACGTTGGAAGGCGAATGCAGAAATC

ACGCCAGAGATGATTTATAAATGGCTGACACCGAATTTTCCTCTCACAGA

CGAAGACCTCTTTAAACCCGCTTTCACCGAGCTCTTGTACGGGATTAGTT

CCGACGAAGAAACGGCGCACAAGTTTCTATGGACTTATCTCAGAGGGGAC

TACGGCCGAATTGGGCAGGTTCAGTTTACTTCGGCCGGCTATGATCATAT

CGATAAACAAGCGGTCCGGAAATTTACCAACTGGGTGAATGTTGAGCACC

CTAGATTCACAGTCCGAGAATAA

>Hp_Contig2080.1

ATGCGTCCCCCTATCATACTTCTCGCTTCGACCGCAGTTTCGGTGTGCAT

TACTGGTGCCCCGGAAGTCACCAACTCCTCCACGAAGGAAGCCGACATCT

CGTCCAGTGTTCCATTCGTTGGTGATGACTCAATCAACGCTCCTGCCAAT

CGACGGTTGAGGGCACACGACGCCGTGGGCGGTGAAGAGAGAGCTCCAGT

GCCGTCCTCATTGACCACTGAGATTTTGAAGAAAACCGAGTTAGCCGTGC

GTAAGCTCTACGACGGTACCAACAGGCCCAACAGTCTTAGCATGCACGCT

TGGCGTTCAGTGATAAATGCGGATAGTAAAATAATGAAGCATGTGATCGC

GGTGGCTGAGAGAAAGAACCAGCGCGTGCCTCAGCAAACCCCGAACGGGG

AGACAACCGGAGACGATATGTCCGGTTTGGTTCTAACGGCTCAAGCGAAC

CATCTCCAAGGGGAAATCGACAAACTACCTATCATGAAATGGATGACGGG

TAAAACCACCGGGGAGCTCGTCGACGTCTTGAAAGAACACAAATTTTATA

ACACGTTCTCTTACACACGGGTGCAAGCGTTGACAGTTGTTCTCGAGAAC

TTTAACGACGCGATGAAGACGAAGATCAAGCTGTTTGACACGCTTGTAGG

AGTGTATGGTGGTGTGGCCAAATACGCACGCGTTTTATCGATCGCGAAGG

CAGGTGTTCTTACTGGCAAGATGATATTGCCGCTCCAGACTGAGCTGGTC

AAGAGCCTCCATTGGGAAACACTTGACGACCTTCTGGTCCTCCTAAAGTT

GGACCTGAAGCGCGACTCCTTGACATACGAAGCGCTGGATACGGTAGTCA

CATGCGCTGCTTTGCGTTACAAGTTGTCAGTAGATGATGCCCTTTATGCC

ACGTTTACGGGTCTACGAGCCATGTATACTGACGAAATCGTTAAAGACGC

GATTAAGAAGGGAAGGGGTGAAATCATATCTGCC

>Hp_Contig214.3

ATGCGCCTAATCTCGTTTGCACTTGCAACGTCCACTGCGATCCTGGCACG

TGACACAAACTCTTCGAGGACACGTGGCTCCACAGTGACGAACGCCAGCC

TCCCGGCCATCTTTCGTTCCTCCGTCGGCAACCATAATGATGTCGTTGTC

AAACGGCTATTGAGAGCACGCGAAATCGCAGCCGATGAAGAGAGAACGCC

AAGAAAACTACCTTCGTTCGACAAAGTGATTAGTGAGCTGTTTGCTACAC

TGCATGTGGAAGAGACATACCCGCCTGACCTCGGTAAAAAAATTATAAAT

AAGTTGCGAACATTCGATCGGGAGGCCATCAAGCACTACTACGAGAAACA

ATATGAGGATCCCATCATGGCGACTAAAAAGCTCATAGAGGCGAGTAGCC

TCAAACATCAACGAACGCATGGGCCATTTGACATGGAAATGTATCGAGAG

TTCTATCATGACCATCTTCATAAAGAGACCTGGATATCGCACTGGGTGCA

AGATGGGTTGCACAAAGCCGAACTGCATCCAAATGTCGTCTTCAAGATGA

TGATCAATGCTGAGAAGCGACCAGTACTGGGCGCTGATTCACGAAGTCTT

TTCGCAACCACGGACCTGGAAGCTTTGCATAAGTACATCGAGCGATTCAA

CGAAAAAGAAAAAAGCAGAACTCCGGCATCGTTGCGACAGACTCTGTCAT

ATTGCATTCGTGACGAGGCCGGACTCGCATCGTTCTTGTCGATTGCGAAG

CAGAACAGTATCAATGCGCCCTTTGTGTGGAAGGAACAACATCGGTTGTT

TATGGGGTGGATAGGCCACAGAAAGACCATCGACCAAGTTGCGGATATGA

TGAAGATTCCAGGACAGTGGAAGGACGCTAAAGCATGCCCATTTTTGGAC

ACTTTGATCGGATACGTTACGGTATTCGCCCAAACGTACCCTGCTGCATC

AACAGACATCGTCTCATGTCTGGTGATTAAGTTTGGCCACCTGTACGCTG

CAATGTTGATTGGAGAAGCGAAGGAGGTGAACATAGATGTTTTCGCCGAA

CTGGAGAAAGTGCTCTTTCAGAGCTGGACGAAAGGTGGCACCAATCCCTT

GAATTTCGATCAAGCGGACTTCTTCGCGGAAATTACTGTTGGTGCGGACG

ACAAGGCGTTGATTCGTGAACATTTTGCTGAGCACTATCGTAAAGAAACG

CCGAGCCATCTTATGTTGACGCTCAACTAA

>Hp_Contig258.6

ATGCGAGGAAGATCTTGGACCGTCTGCATCCTGACCATGCCACTTTCGAA

TTTTCCAATGGCTGGCTTAGTTCCTTCAAGGCCCGTCATGGGATCCGTTC

GTGTTGCCGCTTTGGAGAAAGAGGATCTGTCAATATGGCGCTTATTGAGA

AGGAGCGGCCCAAAATTCGGGAGGCCTTGGACAAGTACGCGCTTGCGGAC

GTGTACAACATGGACGAAACGGGACTGTTCTACAGGATGCAGGTACAATT

TGATTCCCTGCATGATTTATATTATGATACATTATAATATTATTCATGTT

ACCGGGCCGACCATTCCCTTACAACCAAGCGGCTGGAAGGTCGGCAACAG

CACAAAGAGCGCCTCACTATCTCGGTTTGCTGCAATGGGGACGGTTCTGA

CAAGCTTCTTTTGTGAATCATCGGACACTTCAAGAAACCTCGATGCTTCA

AGAACATTAATATCAACAATCTGGGTTGCACCTACCGCAGCAACGCAAAA

GCATGGATGA

>Hp_Contig388.1

ATGCGATTCATCGCCCTCACCCTTGTTGCGTCAACCGCTCTTCTGCCCCG

AACTGAGAGTGCCACGGAGGCTGCTGGTTCCTCGACGACGGATCAACGGG

ACGCAGCTGTTGTCGTCCACGTGACCTCCGATTACTACGGCTCCCTTTCG

AGCAGGTTGTTGCGGTCGCAAGTAGGACCTTCTGAAGTTCTAGAGTCCGC

GATCAGTGCGGTTGCCAGTCGTATTAAGCCAGCCAAATCGTTCTTCGGGA

TCGGGACCGCGCGCGTTAGACGGTCGATATTTCATGTGGCCGGAAGGTGG

TATAGCCATGGAGATCAAGGAGCCTGTTATCAAAGCTGA

>Hp_Contig399.11_ATR39-1

ATGGTCAAGTGCACTCCTCTCCTCGCGCTCACAGTCATTGTGTCTGCTGG

CAGTGACGCTCTTTCGGACCCAACAGTCAAAAGACTGGCGAAGCTGGCGA

CGATAAATCAGGCCCCAGCCACTCAGTCGAATTCGGACAGTAAACGTGTG

CTTCGAGCGTCCGATGTCCCCGATGAAGTCGCTGCTGGTGAGTCAAGGTC

ACCGAAATCACTGTGGCCATGGGAGGTAGAGGACAAGCTAGCCCCTTTAA

AGGAAAAGCTAATTTCCACGTCGGCCGATGACCTCGAGGCTGGAGGATCA

GCAAACAAAAATGCTCTCCCCATGATATGGCGCTGGCTTTGGCAGAACCA

GATCGAAACCAAGAAGACGCCAATAGATGAGATTTCGAAGGAGGTACAGA

CTGCTATGGACCTAATTTCAAGCCAAGCAACTCATGAAGAACTTAACAAA

GCGGGTGTTTCAGTATCTGATTACGTCAAAGCCCTGAGATTTACATTGTC

GGATATAGATGTGGTGAATAGAGGCATGGAATACAACATACATCTGGGTA

ACAAATAA

Hp_Contig399.12_ATR39-2

ATGGTCAAGTGCACTCCTCTCCTCGCGCTCACAGTCATTGTGTCTGCTGGCAGTGACGCTCTTTCGGACCCAACAGTCAAAAGACTGGCGAAGCTGGCGACGATAAATCAGGCCCCAGCCACTCAGTCGAATTCGGACAGTAAACGTGTGCTTCGAGCGTCCGATGTCCCCAATGAAGTCGCTGCTGGTGAGTCAAGGTCACCGAAATCACTGTGGCCATGGGAGGTAGAGGACAAGCTAGCCCCTTTAAAGGAAAAGCTAATTTCCACGTCGGCCGATGACCTCGAGGCTGGAGGATCAGCAAAAAAAAATGCTCTCCCCATGACATGGCGCTGGCTTTGGCAGAACCAGATCGAAACCAAGAAGACGCCAATAGATGAGATTTCGAAGGAGGTACAGACTGCTATGGACCTAATTTCAAGCAAAGCGACTCATGAAGAACTTAACAAAGCGGGTGTTTCAGTATCTGATTACGTCAAAGCCCTGAAATTAACATTGCCGGAGGTAGATGTAGATGTGGTGAATAGAGGCATGGAATACAACATACATCTGGGTAACAAATAA

>Hp_Contig4150.1

ATGCGGATTACCAGCGTCTACGTGGCCATTGTTGCGGCATCTCTTCACGC

GATTGGATCTGCCGTGTCCACGGCCACGAACGTCGGCGCGACCGTGCTCG

AGAACAAGATACCCGCAGCAATTGCTCCGTCCCTTCGTACGAGTGAAGGA

CGAAGTCTACGGACTGAAGAGAAGCGCGGTCAGAGCGACCTGGGAGAGGA

AAGGGGGCGCGGGAATCTACCGACTACCGATAGTTTTAGTGCATCCCTAA

AACGCTTCATTCGCGCCCTGTTTTGCATGGGATCGAAAAAAGGAATGACA

CGAACCAAGTCGTCGGGTTCTTCCATCGTGTCGGGTCATGGTAGG

>Hp_Contig450.9

ATGCGTCTTATCTGTGTCTTGCTACTCGCCGTCGTCTCCCAGATGACAGT

GTCAAGCTACGTTGCGGATGCCAAGGACGTGACAAAACCCGCTAACCAAG

GGACCGGATCCGAGCAACTGAAGGCCCGTCGGTATCTCAGGGATGACAAA

GGTACATTTGGCGATCAAAACACCGCGATGAACAACAAGGACGAGGAAAG

GATATTCGAAGGGGTTTTTAATAAGTTGATGGCGCTCTTTCATGGGAGCA

TCGACGCAAAGCAAGTGAAGCCACTGATCCAAGAGACTCACATTCTCGAA

GAGAATGTGGCAAATCTCCAGGCTATTCCGACAGCCTCCAAGAAGGGTCT

CTCGCAATTCTTCGCTGAGAATCCTGCCATTAAGAAGGAGATTATTGTAG

CGGGAGTTCTTTTGTCTTTGATCGTCGCAGTTCCGCTGACTGTTAAGTCA

TTTTATCCAGCTTAA

>Hp_Contig463.5

ATGCGTCTGACCTACACCGCATTGGCCACTCTGGCTGCTCTGCTGTCTTG

CAGCGACTCAGTCCCGACTGCTGGTAATCCGAAGGCTGTTCTTAAGCACG

ACGTGTCTCTTGTGGCTCGCGGGACGGGTGGCGACGACAACGGAGCAGAC

GGCAAGAGGCTGCTGAGGAACGCTCATGCGCAAGATGAGTCGCAAGATTC

TCAACTGTTTGAGGAAAGATCAGTACATGTACCAGAGAAGGCGACTGTGG

TGGTGGCGTCCGGGGTTGCTCATGTCATCCCTTCGGAGGCAAGTACGATC

GCGAAAAAGGTGGTGGAAGCGAACACTCATGTGAAAGACGGCGCGGGTCA

AGAGAAGTACTGGTTGTTGACGAAGCTCGAGTCGTTCAAGAAGAAGCTGA

TGGAGAGCCGCAAGTTCCAGGCAATCATGGAAAAGCTTAACACGTTTCTA

GGCCGCAAACCGACGACCACGGCTGGGAATACCAACACCGTGAAAGACGA

GGGCAAGGCGACAGCGAATGGTGGGACCCACACTACGCATGATGACAAGC

CACCGGTGACCACAATAATTCATCCCGAGGCTGGCGGCCAGTCAAAATTT

GCTTCGACTAGAGGCGCCGTGGCGGAGAGGATGAAAGATGCGACCAAGAA

GACGCAGGACAAGGTGGCCGAATGGAACAAGAAGCTTGAGCAGTACAAGA

AGAAGGTGAAGGAGGACCCCAAGTTCCAGGCAATCATGGAAAAGCTTAAC

ACGTTTCTAGGCCGCAAACCGACGACCACGGCTGGGAATACCAACACCGT

GATAGACGAGGGCAAGGCGACAGCGAATGGTGGGACCCACACTACGCATG

ATACAAGCCAGAGATGA

>Hp_Contig550.2

ATGCGTCTGTCTGCTATCCTGCTCTTAATTGTGGCTCCCCTGCATCTTTG

TGTCGGCGAGGTGGTCCTGATCCCTGCGACGATGGAGAACCCACTTCTTC

GTTCGGCTCCTTCTACTAATGCTGATGCACGTAAAGGAGCCCGATCACTG

CGAGCGCTAAATTCGGCTGGCATCAGCCAGCTGCTGGAGCCGTTCACCAC

AAAGGTGAAATCGTTTGTACCAAGGACTGCTGCATACGCTGCAGCAAAGC

AGGCAAGAGTTAAAAAAGCAGCGGCCGCACGGACAGCTCAAATTGAGCAA

AAATCAGAAGAGAACCAGGCGGCGCTCTTGAAAATGGGCTTAGCTGCCTT

CGGTCAAGATGTTAGCTCGGCGATAGTGAAAGCGAACAGCCAGAATTCGT

TTTTTCAACGCGACGGCAGTTTTTTTCTAATCCTTTTCAAAAGGGGGAAA

TCCGTCGATGACCTTACAGGGATGCTCGAGAGCGCCTGCCAAACAAGTGA

TATATCGGTCGAAGTCGCGGTGAGGGATACCGTCCGCCAATACAAACTGT

ATCGAGAGGACCCAAAAAGGCGTGAAATATCTTTTCTCTTGGCGAATCCG

GCATCG

>Hp_Contig750.2

ATGCGTATCCACGTTCGTGTCCTGCTGGGTGTAGCCGCTCTAGTCGTGGG

CATCCACACTACCGTAGCATTGGTGCGGACGGAGACTACGAATGAAGACA

CCCAGCTATCTGGACGCATTCATTCAACCGGGGATAACGACGTCTCCTGC

CGGCGCTTTTTGCGGACGAACGACGCGCATGCTAAGGACGAGGAGGAAAG

AGTTGACACCAAAGCAGTATCGGCATGGGTGCAAAAGGTTCTCAGCAAGC

TAAAAACTTCTGCACTGATTGATTGGCATCTGATATTAGACCACGACCCC

GGATACGTGAGAGGCAAATATCCTAACGACAAAGTGCTCGGTGATAAGTA

CTATGCAAGATGGGCGGATATTAAATATCGTAAC

>Hp_Contig904.6

ATGACCAAGTGCTCCCTACTTCTCGTGCCCTTCCTCGTCGCAATTGCTGT

CAGTGACGCTCTGCCAGCTCGCGTAGCCGGCACGCTGCCACAAAGCGCAA

CATCGGTCCAAGACAAGGCTACTGAGTCGACCGTGAGCGGCAAACGAGCG

CTTCGATCAAAGAAAGACACGAAGGGTGCGGCTGATGAAGAAAGAGCGCT

ACTTAGTCCTTCGATACTGGAGCCGTTGAGCACCAAGATGAAGAGCAGCA

CTGACTGGATGGCTCAGACGAGGAAAGGAGCTTCTTTTAGCGTGTCGGGG

GTGTCGCAGAAAGAGCTGGGGGAGTCGAAGGATGTCGTGGTGAGATTGGA

GAATCTACAGCGCGACTTCCAGAAGAGTACGGAACAAATGACAATTGATA

TGGCGAGGGATCTGACGTTTTCGGGGGCAACTCGGAAGGTGGGACGGGGA

ATTACTTGGCTCCCGTTGAATCGTGTGTTGCAGGACAGAAAATATCGTGA

ATGGCTGGAGACCATGAGCAAGAGGGATGCCTACGCTGCTAAGCTGGAAG

AGTTGATCGCCGCGGCGAAAAAAAGGGTAGACGGCATCAATCCGGCGATT

TAA

>Hp_Contig96.3

ATGCGTCTGACCTACACCGCATTGGCCACTCTGGCTGCTCTGCTGTCTTG

CAGCGACTCAGTCCCGACTGCTGGTAATCCGAAGGCTGTTCTTAAGCACG

ACGTGTCTCTTGTGGCTCGCGGGACGGGTGGCGACGACAACGGAGCAGAC

GGCAAGAGGCTGCTGAGGAACGCTCATGCGCAAGATGAGTCGCAAGATTC

TCAACTGTTTGAGGAAAGATCAGTAGCTGTACCAGAGGCGGCGACGGGGG

CGGCGTCCGCGGTTGTTCATAGCTTCACTTCGGAGGCAAGTCCGATCGCG

AAAAACGTGGTGGAAGCTACCACTCATGTGAAAGACGGCGCGGGTCAAGA

GAAGAACTGGTTGTTGACGAAGCTCGAGTCGTTCAAGGACTGGGCGAAGG

AGCTCTCGTTCATCAAGGGTATCATGAAATGGTGGAAGCGCTGGATCCAC

AACCCCAACCACGTCGATAAGAACGCGAAGCCGGTTTCATCGACCCCTTC

GGTCGAAGGCGAAAAGATTACGGAGAAGGAGGCCGAGGTTAAGAAGAGTG

CTACGGCCGATAAATCCCTGCCGCCGCCGCCGGCGTATACCCCACGTCTT

TCCGAAGGTAACGTCAAGACTGCTGGCGAGACCAGCAAAGTTCCCACGGA

CAATGGCAAGCTGCCGGTAGGTACTGTTACAAAGACCGAGGGCAAGAGTA

CGGTTTCCAGCCAGAACGAGAAGCCGATAATGACTGCTGGAACTCATGGT

ACCGATTCAACGAAACCTCAGGGCGCCGGCAACCCGTCCAAGAACACCGA

GGGTGGAGTCCCTTTAAAAATTGATGCTCCTGTGAGCACTCAGGGTCACA

AGGATGGC
